# Ants evade harmful food by active abandonment

**DOI:** 10.1038/s42003-023-05729-7

**Published:** 2024-01-12

**Authors:** Daniel Zanola, Tomer J. Czaczkes, Roxana Josens

**Affiliations:** 1 Laboratorio de Insectos Sociales, Departamento de Biodiversidad y Biología Experimental, Facultad de Ciencias Exactas y Naturales, Universidad de Buenos Aires, IFIBYNE, CONICET, Ciudad Universitaria (C1428EHA), Buenos Aires, Argentina; 2 Animal Comparative Economics Laboratory, Faculty of Biology and Preclinical Medicine, University of Regensburg, Universitaetsstrasse 31, 93053 Regensburg, Germany

**Keywords:** Animal behaviour, Entomology, Social behaviour

## Abstract

Invasive ants, such as the Argentine ant, pose a severe economic and ecological threat. Despite advancements in baiting techniques, effectively managing established ant populations remains a daunting challenge, often ending in failure. Ant colonies employ behavioural immunity against pathogens, raising the question of whether ants can collectively respond to toxic baits. This study investigates whether ant colonies actively abandon palatable but harmful food sources. We provided two sucrose feeders, each generating a new foraging trail, with one transitioning to offering toxic food. Six hours later, ant activity on that path decreases, while activity on the non-toxic food and the trunk trail remains unaffected, excluding factors like population decline or satiation as reasons for the activity decline. Laboratory experiments confirmed that ants remained alive six hours after ingesting toxic food. Ant presence remains low on the toxic food path for days, gradually decreasing along the nearest section of the trunk trail. This abandonment behaviour minimises the entry of harmful food into the nest, acting as a protective social mechanism. The evasion of toxic bait-treated areas likely contributes considerably to control failures. Understanding the behavioural response to toxic baits is essential for developing effective strategies to combat invasive ant species.

## Abstract (web summary)

Ants exhibit a behavioural strategy of actively abandoning toxic foods and gradually avoiding the area to protect the colony, reducing the entry of harmful foods. This behavior threatens invasive ant control measures, since the ants abandon the baits.

## Introduction

Invasive alien species pose a major global problem, profoundly impacting natural and anthropized ecosystems. Ants have emerged as especially damaging^1,2^. Over 200 ant species have established populations beyond their native ranges^1,3^. Of these, 19 are listed in the IUCN’s database of invasive species, with five species ranking among the “100 worst invasive alien species”^4^. The economic cost of invasions is staggering, estimated at 52,000,000,000 US dollars^5^, affecting agricultural production, causing infrastructure damage, disrupting electrical equipment, and potentially acting as a disease vector in hospitals^1,6–9^. However, their ecological effects may be even more profound^10^.

Invasive ants strongly displace native ant communities^11^, cascading up trophic levels and affecting native vertebrates, including birds, reptiles, and amphibians^12–14^. These invasions disrupt ecosystem functions by altering trophic web dynamics, modifying nutrient cycling, and diminishing pollination services^15–17^. Improving our understanding of invasive ants, and addressing the challenges posed by them, should thus be a priority for conservation efforts. This is doubly true, considering that around two thirds of eradication attempts have failed^18,19^.

Among invasive ant species, *Linepithema humile* (the Argentine ant) stands out as a particularly notable invader (see http://www.issg.org/database), especially in Europe, where it is the most important invasive ant^20,21^. Originally native to South America, it has been introduced globally^22^. Although initially recognized as an urban pest^23^, the Argentine ant’s adverse impacts extend well beyond urban settings and permeate natural and agricultural systems^24–26^. In invaded natural areas, the Argentine ant can profoundly impact native fauna, leading to disruptions in essential ecological processes such as seed dispersal^27,28^ and pollination^29,30^, thereby exerting negative effects on native biodiversity. Their unicolonial population structure allows a high population density, and therefore efficient resource utilization. Argentine ants thus gain a competitive advantage over other ant species^31,32^, leading to displacement of native ants, disruption of invertebrates, and even adverse effects on vertebrates^22,33–35^. In agricultural systems, Argentine ants are associated with outbreaks of phloem-feeding hemipterans, affecting the growth and productivity of host plants^24,36^ and disruption of the activity of the natural enemies of these agricultural pests^37^.

Traditionally, control methods for Argentine ants have relied on contact insecticides acting as barriers, which only offer partial suppression and have limited efficacy against the queens and brood sheltered within the nests^38^. Furthermore, the rapid degradation of chemical barriers necessitates frequent reapplications^39^. By contrast, toxic baits have several advantages: They are less ecologically damaging, since they require smaller amounts of insecticide and thus minimize unwanted effects^40^. Locating the nests is not necessary, since the ants locate the bait, return it to the nest, and distribute it to the rest of the colony, including the queens. As invasive ant species commonly show mass recruitment, they often monopolize resources, which in the case of toxic baits minimizes impact on non-target species^41,42^.

Thus, modern control methods predominantly rely on toxic baits, with liquid sucrose baits being especially attractive to this species^43,44^. However, in spite of impressive technological innovations, such as the development of hydrogel beads to broadly deploy liquid baits^42,45–48^, eliminating established populations of Argentine ants has proven challenging, with only limited success reported^41^. In field studies, baits often fail to control Argentine ants for more than 60 days, and there is often a resurgence of ant populations thereafter, or reinvasion after treatment by ants from nearby untreated areas^41^.

The effectiveness of toxic baits depends heavily on the attractiveness of the bait to foraging ants so as to ensure sufficient, sustained consumption^43,49–51^. However, the acceptance of toxic baits by ants may be influenced by changes in the availability of alternative natural food sources^35,41,52,53^. Therefore, the acceptance of a toxic bait observed in a specific situation may not necessarily reflect its acceptance in other scenarios^54^ or the effectiveness when used in a control program. Formulating toxicants into consistently acceptable baits has proven to be one of the most challenging aspects of invasive ant control^43,51,55^.

While much research effort has been devoted to developing attractive and effective baits, there is a paucity of studies examining the potential behavioural mechanisms ants may use to evade toxic baits. This may be an important oversight. Ants are known to deploy a variety of behavioural strategies for avoiding dangerous substances and situations. For example, ants, including *Linepithema* ants, abandon foraging paths or food resources when phorid parasitoid flies are present and tend to avoid areas or times of the day frequented by these flies^56–58^. Likewise, ants begin to avoid paths associated with mortality risk, while continuing to forage in safe areas^59,60^. Leaf-cutter ants quickly learn to avoid leaves bearing fungicides which damage their fungal gardens^61,62^. Dangerous substances, such as sticky surfaces, may be covered with dirt and debris, to make the areas safe^63,64^, and ants avoid moving into nests which contain conspecific corpses^65^. Indeed, ants display a wide variety of social immunity behaviours to avoid disease. Spore-laden pupae are disinfected, or if too infected, destroyed^66^. When ants detect a pathogen in the environment, they modify the interaction network of the colony so as to isolate foragers from the critical queens^67^.

Here we ask: do invasive ants possess behavioural mechanisms, potentially similar to social immunity behaviours, which allow them to evade toxic baits? Specifically—can ants abandon otherwise palatable toxic bait?

## Results

### Palatability test

We assessed the palatability of the harmful food, (hereafter referred to as ‘toxic bait’) for our upcoming trials, specifically evaluating whether ants drink it. We presented individual drops of sucrose solution (positive control), sucrose solution containing our toxicant (3% boric acid), and sucrose solution with c. 0.26 g/L quinine (a distasteful substance which is expected to be rejected, as a negative control) at different locations alongside *Linepithema humile* trunk trails.

Almost all ants that touched the drop of sucrose solution with their antenna fed on it (98.4% ±0.6, mean ±s.e.). In total 399 ants over 19 drops were tested. A similar result was obtained with boric acid-sucrose solution (toxic bait) (97.8% ± 1.2), based on 167 ants over 9 drops. However, the sucrose-quinine solution was accepted by only 15.7% (±6.6) of the ants that touched the drop (from 206 ants over 9 drops). Therefore, we can confirm that this toxic bait is palatable for this species under the natural conditions of our study.

### Experiment 1: Day-wise dynamics and spatial extent of trail abandonment

To investigate whether ants employ a behavioural strategy of abandoning foraging trails in response to toxic baits, we established two new foraging trails by installing two bridges, both initially offering sucrose solution. Once foraging was established, and foraging activity equalized between the two foraging trails, we recorded the ants’ activity at this point as a baseline measurement (referred to as ‘time 0’). Immediately after baseline recording, we introduced permanent feeders, one containing the same sucrose solution and the other containing sucrose with added toxicant (toxic bait). We monitored activity levels on both the toxicant and sucrose bridges, as well as at various points along the trunk trail both in the afternoon (averaging three recordings at 4 pm, 5 pm, and 6 pm) and the morning (averaging three recordings at 9 am, 10 am, and 11 am) over a four-day period (referred to as ‘time 1’ through ‘time 6’). For example, time 1 encompassed the average activity recorded one, two, and three hours after the access of the final feeders. (For a schematic timeline of the experiment, see Methods).

#### Foraging trail

**Fig. 1:**
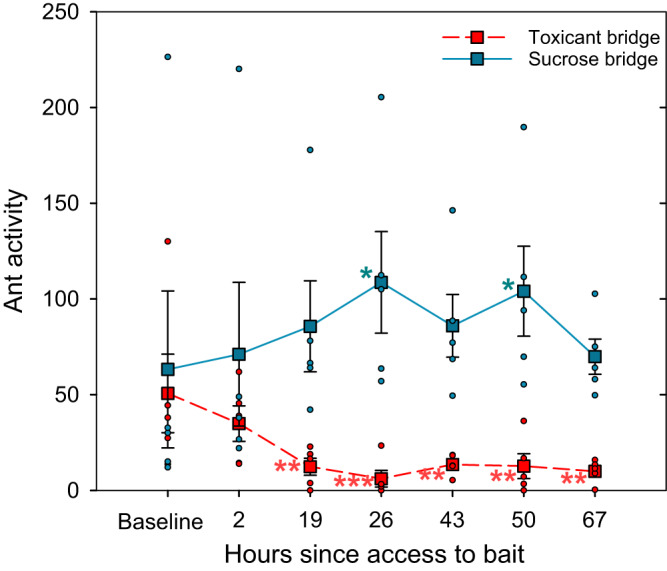
Temporal dynamics of ant foraging activity on bridges over days. Ant activity measured as the mean number of ants crossing a line on the bridge (foraging trail) toward the foraging arena over a minute, in two bridges connected to the same trunk trail, separated by at least 7 m. Each bridge had its own baseline measured at time 0, when both offered drops of sucrose solution. Immediately after the baseline recording, the feeders were opened, offering sucrose with a toxicant on one bridge (Toxicant bridge, in red, dashed line) and sucrose solution on the other (Sucrose bridge, in blue, solid line). Activity is shown as a function of the hours after feeders were opened, i.e., when the ants began to forage on the toxic bait. Squares are means ± SE. Circles are data of each replicate. Significant differences are shown for each bridge by comparing the activity at each time to the baseline of the same bridge. *n* = 5. (*p < 0.05; **p < 0.01; ***p < 0.001; no symbol: no significant differences).

Both bridges initially showed similar activity levels when offering sucrose drops (baseline at time 0: estimate = -0.07; p = 0.79). There is a clear interaction effect between the solution offered on the bridge and time (from time 2 to 6) (see Supplementary Material). Activity begins to diverge between both bridges within the first 3 hours after the feeders were opened (time 1: estimate = -0.62; p = 0.026), with a slight tendency to increase in the sucrose bridge and to decrease in the toxicant bridge. Starting from the next morning (time 2), this difference in activity between the two bridges becomes much more pronounced, and persists throughout the following days (see Fig. 1 and Supplementary Material).

For the toxicant bridge, over the 1- to 3-h interval after the toxic bait tubes were made available (time 1), activity did not differ from the baseline activity of that bridge (time 0 vs. 1, estimate= 0.34; p = 0.647). In the subsequent period, time 2, corresponding to the next morning (i.e., 18 to 20 h after bait availability), activity on the toxicant bridge significantly decreased compared to its initial activity (time 0 vs. 2, estimate=1.4; *p* = 0.004). The activity on this bridge remained at low levels, with slight fluctuations, all significantly different from its baseline (Fig. 1, red).

By contrast, for the sucrose bridge, activity at times 1 and 2 did not differ from its initial level. Then, the activity increased slightly and remained high throughout the experiment, with fluctuations that were mostly not significantly different from their baseline (Fig. 1, blue).

The reduction in foraging activity observed at the toxicant bridge occurred within the first 18 hours of access to toxic bait. The mean activity recorded at this time was 12.4 ants per min, which represents 24.5% of the mean baseline. In other words, at time 2 the activity is 75.5% lower than its mean baseline, and remained at these levels with minimal variation (73% to 88%) throughout the duration of the experiment.

As activity at the sucrose bridge never drops, we can exclude satiate or a reduction in foraging motivation as explanation of the decrease in the toxicant bridge.

Considering that activity on the toxicant bridge was lower than baseline by time 2 but not by time 1, we wondered whether any trend within the time range encompassing time 1 could be observed—remember that time 1 is composed of three measurements, one hour apart. When analyzing each of these 3 h separately to determine if they differ from the baseline (time 0), we observe that there is no difference from the baseline during the first two hours of bait consumption. However, after 3 h of bait consumption, a trend emerges, showing a marginally significant decrease in activity (15hs-18hs: estimate= 0.61; *p* = 0.057). As expected, during this period, the sucrose bridge, which offers sucrose, exhibits activity very similar to its baseline (Supplementary Fig. S4).

#### Trunk trail

To study activity dynamics on the trunk trail, activity was measured at different locations: the toxicant bridge site (0 m), the sucrose bridge site (~7 m from the toxicant bridge), and 2 m and 4 m on each side of the toxicant bridge site (averaging left and right at each of both distances).

**Fig. 2:**
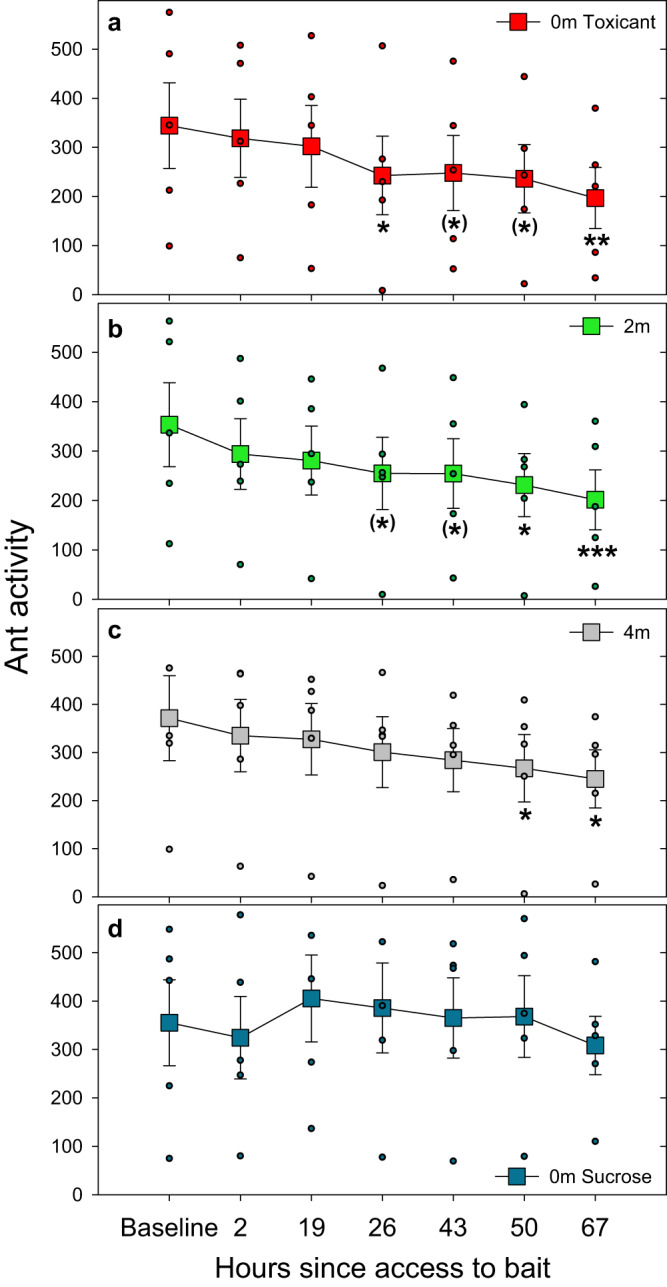
Temporal dynamics of ant activity along the trunk trail at multiple locations over days. Activity measured at different locations along the trunk trail at (**a**) a bridge offering a toxic bait, and at 2 (**b**) and 4 (**c**) meters either side of this (2 m and 4 m; right-left averaged) and by a sucrose bridge offering unadulterated sucrose over 7 meters away of the toxicant bridge (**d**). Squares are means ± SE. (n = 5). Circles are data of each replicate. Significant differences for each location comparing activity at each time point to the baseline for the same site are shown: *p < 0.05; **p < 0.01; ***p < 0.001; no symbol: not significant; ^(^*^)^: 0.05 < *p* < 0.087. See also Sup. Mat. for more details.

There is an interaction effect between sites (at least one) and time (see Supplementary Material). Figure 2 shows that at time 1 (between 1 and 3 h after the feeders were made available on the bridges), the activity of the trunk trail did not vary at any site compared to the baseline activity of each site. This baseline activity refers to the activity at time 0, which was prior to the placement of the bridges.

At time 2 (which corresponds to the morning of day 2), activity also did not differ at any location along the trunk trail. At time 3 (corresponds to the following afternoon, day 2, approximately 25 to 27 h after the feeders were opened on the bridges), activity began to significantly decrease only at the toxicant bridge site (0 m; time 0 vs time 3: estimate= 0.37; *p* = 0.048. Fig. 2), and marginally significant within a 2 m radius (2 m; time 0 vs. time 3: estimate= 0.34; *p* = 0.067). Activity remained similar to the corresponding baselines beyond 2 meters of the toxicant bridge site at that time. The situation remained similar in time 4. The low activity persisted at the toxicant bridge site and 2 m, resulting in significant or marginal differences from baseline thereafter. Only from time 5 onwards (49 to 51 h of foraging on the toxic bait) did the decrease in activity became significant beyond 2 m (4 m; time 0 vs. time 5: estimate= 0.36; *p* = 0.042).

Finally, at time 6 (morning day 4), activity showed higher significant differences in the area around the toxicant bridge, extending up to 4 m on both sides (4 m; time 0 vs. time 6: estimate= 0.43; *p* = 0.013).

In contrast, activity on the trunk trail at the sucrose bridge site did not vary at any time relative to the baseline. It is worth noting that this bridge is located on the same trunk trail, over 7 meters away from toxicant bridge (see also Supplementary Materal).

Also important is that while activity directly on the toxicant bridges significantly decreased by time 2 (Fig. 1), activity on the trunk trail in the vicinity of these bridges (toxicant bridge and 2 m) remained unchanged at that time, and only began to exhibit a slight decrease starting from time 3 (Fig. 2). This demonstrates that the approximately 75% decline in the toxicant bridge activity at time 2 cannot be attributed to a decrease in the ant population. While activity on the toxic bait decreased by around 80% after 6 h, activity on the trunk trail ultimately decreased by a maximum of 43%, and did so only by the end of the study, 3 days after the toxic bait was presented. Indeed, at more distant locations (4 m from the toxicant bridge), activity only began to decrease at time 5, half way through day 2. This demonstrates a progressive abandonment that initially starts at very close proximity to the bait and gradually expands outwards, involving the trunk trail.

### Experiment 2: Hour-wise dynamics of trail abandonment

Experiment 1 revealed that abandonment of foraging trails at the toxicant bridge had already occurred 18 hours after the toxic bait became available. In this experiment, we examined abandonment dynamics in a similar manner but with higher temporal resolution during the first day, recording ant activity on both bridges (sucrose and toxicant) every hour (time 1 to time 8). Here, again the initial measurement was with both bridges offering sucrose solution (time 0 = baseline).

#### Foraging trail

**Fig. 3:**
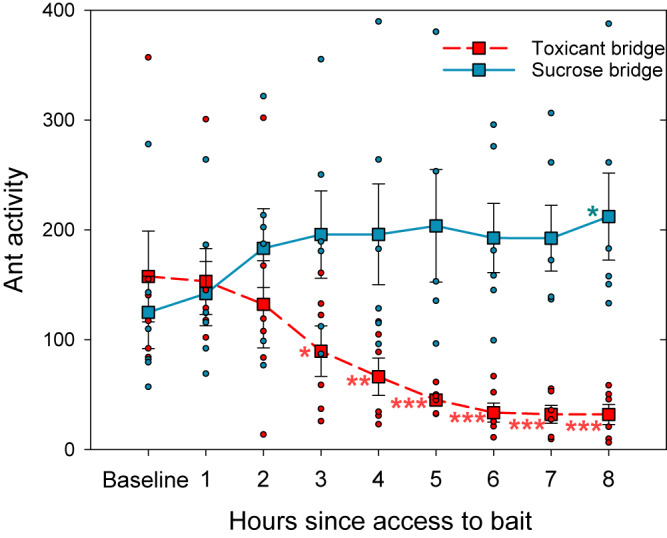
Dynamics of toxic bait abandonment over 8 hours. Ant foraging activity on the bridges as a function of time (h) over 8 hours. Baseline ant activity was measured at time 0 with both bridges offering sucrose solution. Immediately thereafter, the feeders were opened, offering sucrose solution in the sucrose bridge (in blue, solid line) and the toxic bait in the toxicant bridge (in red. dashed line). For each bridge the activity of each time (from time 1 to time 8) was compared with the corresponding baseline. Squares are means ± SE (*n* = 6). Circles are data of each replicate. Significant differences are shown for each time compared to baseline; for the sucrose bridge above the curve and in blue, and for the toxicant bridge under the curve and in red. (*p < 0.05; **p < 0.01; ***p < 0.001; no symbol: no significant differences). See also Sup. Mat. for more details.

There is a strong interaction between the solution offered on the bridge (toxicant or sucrose) and time (from time 2 to time 8) (see Supplementary Materal). Ant activity on the bridge offering the toxic bait began to show a significant decrease after 3 hours of foraging on the bait (time 0 vs. time 3: estimate=0.56; *p* = 0.04. Fig. 3), with a 43% reduction relative to its baseline activity. This reduction increased, becoming more strongly significant over the next two hours, reaching 58% after 4 h and 70% after 5 h. Thereafter, this reduction in activity remained essentially constant, reaching 79% by hour 6 and remaining at 80% for the next two hours measured. Such a reduction in foraging activity never occurred in the sucrose bridge, located on the same trunk trail about 7 meters away. On the contrary, activity on the sucrose bridge tended to increase and remained high throughout the experiment, with fluctuations that were occasionally marginally significantly higher from the baseline activity of that bridge (Fig. 3). Again, this demonstrates that the ants were not satiated, not killed, and continued actively foraging throughout the experiment.

**Fig. 4:**
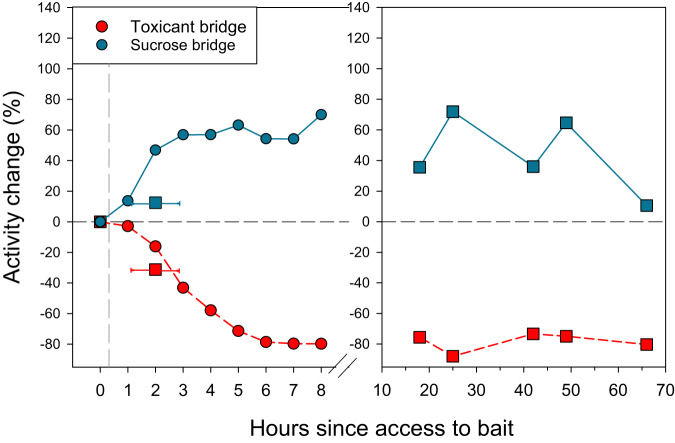
Activity change over time in the bridges integrating both experiments. Activity change is expressed as the percentage change obtained from the average activity for each timepoint with respect to the average activity of its baseline, (and thus, there is no variance). The dashed red curve represents the bridge offering toxic bait after time 0, the solid blue curve represents the bridge offering sucrose solution. Blue shapes represent sucrose solution being offered; red represents toxic bait being offered. This figure integrates two experiments: the left panel shows the high-resolution temporal dynamics over one day post bait presentation (Experiment 2, circles). The right panel shows the temporal dynamics over 4 days post bait presentation (Experiment 1, squares). Note one datum of Experiment 1 in the left panel (square at time 1: i.e., 1, 2, and 3 h since bait access). Each square represents the pooling of three measurements taken 1 h apart. Vertical dashed line indicates the opening of the final main feeders. Horizontal dashed line at zero indicates no change in the activity.

Six hours after toxic bait presentation, an approximately 80% reduction in ant activity on the toxicant bridge was achieved, and this reduction was maintained until the end of the experiment. This value is close to the percentage of reduction of Experiment 1, even though in Experiment 2 the initial activity was much higher than in Experiment 1, achieved by offering sugar for a whole day prior to the experiment. To provide a visual representation of this, Fig. 4 integrates the activity data from both experiments on the two bridges. The activity change is expressed as the percentage change obtained from the average activity for each timepoint with respect to the average activity of its baseline. Thus, the baseline is always zero, values close to zero mean that the activity was similar to its baseline, positive values indicate an increase in activity, and negative values indicate a decrease in activity.

### Mortality assay

To test whether the 80% reduction in activity at the toxic bait bridge may be a result of mortality, we conducted a laboratory test. This involved offering the same toxic bait or sucrose solution to groups of 5 ants from a nest starved for 48 h and assessing their mortality every hour for 6 h.

Twenty-four groups, each composed of 5 ants, were given a sucrose solution, while another 24 groups were provided with the toxic bait (resulting in 120 ants per treatment). At the end of the experiment, nearly all ants survived during the 6-hour measurement period (see Fig. S15). All the control ants remained alive and only 7 ants that had consumed the toxic bait had died after the six hours of the experiment, which represents 5.8% of the total.

## Discussion

We demonstrated that the presence of a toxic bait led to active abandonment of the foraging trail. This abandonment was highly spatially localized and began approximately 3 h after bait placement, resulting in a 70-80% decrease in activity on the bridge after 6 hours. Regardless of the initial population of foraging ants, the percentage decrease was consistent and persisted for several days. Activity remained high on the control sucrose bridge, indicating that the abandonment was not due to satiety or lack of motivation to forage. The trunk trail adjacent to the toxic bridge initially maintained similar activity levels, thus excluding population decline as an explanation. The abandonment gradually spread to the trunk trail but only in the vicinity of the toxic bait, not extending to areas located approximately more than 5 meters away where the sucrose bridge was placed for the period studied. The observed decrease in activity on the toxicant bridge and within the trunk trail cannot be attributed to a population decline, as the employed toxicant does not induce mortality at such a rapid rate. Taken together, these results unequivocally demonstrate a targeted behavioural abandonment of a toxic bait by this invasive ant.

The percentage reduction we report is consistent with what is frequently mentioned in the literature when evaluating the efficacy of bait treatments in various settings, such as urban areas, orchards, and vineyards: mean from week 2 to 11 = 79.4%^68^, 80% reduction after 8-10 weeks^69^, 78% of average reduction^41^; 85% reduction at day 1, averaging 81% during the 1st week^45^. Klotz et al. (1998) reported that, after toxicant deployment inside a building, ant foraging activity had been redirected to the outside^69^. Ant populations around the buildings treated with boric acid bait showed a continuous reduction over the duration of the test, reaching 81% around the treated buildings. Nevertheless, the typical interpretation accompanying a decline in ant presence attributes it solely to the mortality induced by the bait. This interpretation can be ruled out in our study. Note that we do not claim that baits did not kill ants in the other studies mentioned above. It is likely that both mortality and abandonment contribute to the reductions observed in those studies, making it challenging to determine the relative roles of each factor.

Similarly, Boser et al.^70^ examined the control of Argentine ants using sucrose-solution with thiamethoxam (0.006%) soaked hydrogel beads, and reported a gradual reduction of ants beginning 2 hours after bait placement, with a significant reduction by 6 h post-treatment. These authors report that traffic reduction at that time reached 78% - remarkably similar to the 79% reduction 6 h post-treatment we report. It is particularly noteworthy that in the Boser et al. study a different toxicant was used, yielding similar outcomes in both the percentage reduction in activity and the timeframes within which these reductions were achieved. This suggests that the observed effects are not solely attributable to the specific chemical compound employed but rather stem from the introduction of harmful food to the colony.

In our long-term experiment (exp 1), we observed a gradual expansion of abandonment along the trunk trail. After 4 days of toxic bait presentation, the decrease in activity extended up to 4 meters on either side of the toxicant bridge. This suggests that as long as a small percentage of ants continue to forage on the bait, the area abandoned will continue to increase over time. Counterintuitively, the decrease in trunk trail activity only occurred around the toxicant bridge, while remaining unchanged some meters to either side. How can that be, if the ants must pass by the bridge to reach the other side? In several trials, we observed that a secondary path had formed, deviating from the trunk trail and re-joining it a couple of meters after passing the point of contact with the toxicant bridge. A similar behaviour has been reported in carpenter ants, which bypassed a toxic bait by modifying the path of their trail, forming a semicircle about 30 cm away from the bait^71^. It is worth noting that in that study, a small volume (5 ml) of toxic bait was offered, whereas in our current study, our feeder provided about 36 ml of toxic bait. We propose that the volume of toxic bait accessible, the capacity for simultaneous access by ants, and the resultant rate of toxicant ingress into the colony collectively influence the scale and dynamics of abandonment, coupled with the area it encompasses. This could also explain the slight variations in dynamics between experiments 1 and 2. In experiment 2, both the toxicant bridge and trunk trail showed a more rapid decline in traffic (Fig. 2 and Supplementary Fig. S12), possibly due to the higher number of ants foraging on the bait.

Abandonment of food sources or foraging trails in response to parasitoids has already been described in different ant species^56–58^. Ant colonies change their foraging patterns in response to worker loss or to avoid aggressive competitors^72^. However, in these cases, the risk is due to natural enemies, suggesting that rapid detection and behavioural responses could have been shaped by co-evolution. In contrast, the harm caused by a palatable toxic bait may only begin well after consumption, although it is unclear how rapidly malaise begins. Somehow, the ants must associate the bait with its negative consequence, either directly or indirectly. Further research is required to clarify both the mechanism by which harm is detected and connected with a location, and the mechanism by which abandonment is triggered. It seems likely that detection of harm involves conditioned taste aversion, due to malaise caused by the toxicant. Conditioned taste aversion has been reported widely in vertebrates in response to malaise, and in several invertebrates^73–78^, but not found in others^79^. Such aversions can form even when the resultant malaise only occurs many hours post-ingestion^80,81^. Another potential abandonment mechanism is the association between the corpses of ants that died from the toxic bait, acting as a negative stimulus, and cues from the bait itself. This effect has been shown in lab experiments with Argentine ants^82^. However, it is unlikely that this explains the abandonment in the current study as boric acid is considered to have a delayed toxicity^54,55^, while we observed a decrease in activity on the bridge after only 3-4 hours.

We cannot attribute the initial 80% reduction in traffic to mortality. Most studies on mortality are conducted over a span of days, and those involving boric acid consistently report mortality rates below 55% within 24 hours after bait ingestion (delayed toxicity)^54,55^. In contrast, our field data revealed an 80% reduction in activity on the toxicant bridge within a 6-hour timeframe. Hence, it is unlikely that the reduction observed is solely a result of mortality. Nevertheless, in an effort to validate this claim and considering the absence of available literature regarding boric acid mortality within the initial 6-hour post-ingestion period, we conducted a straightforward laboratory test. Remarkably, even without nestmates to receive crop unloading and thus dilute the toxic bait among individuals, most ants which fed to satiation on toxic bait remained alive after 6 hours. Thus, it appears highly improbable that the 80% initial reduction in the toxic bait bridge can be attributed to ant mortality.

*L. humile* has been described as having a high fidelity to well-established trails^83,84^. However, contrary to typical ant behaviour characterized by resource fidelity, our observations in this study showed a distinct pattern. While some ant species display a strong fidelity for stable food sources, often ignoring alternative food sources, we found that *L. humile* ants readily engaged with both sucrose solution and toxic bait, rapidly establishing new foraging trails. They also fully accepted the drops offered in the palatability test. These behaviours challenge the notion that the ants’ reluctance to return to the toxicant bridge is solely due to fidelity to other food sources.

An interesting situation that is related to our results is the finding that leafcutter ants exhibit a delayed avoidance response to leaves that damage the fungus they cultivate. Specifically, once the ants realize that the collected plant material is detrimental to the fungus, they learn to associate its scent (and probably additional plant cues) with harm and cease collecting that resource for several weeks, even if it no longer contains the fungus-damaging compound^62^. In laboratory colonies, the tendency to this rejection starts 6 h and becomes significant from 10 h after incorporation of treated leaves into the fungus garden^85^. In the field the rejection was evaluated and observed at 24 and 48 h, so no information is available with more temporal resolution^61^. Interesting, the item rejection last for 17 weeks.

As the term ‘avoidance learning’ typically refers to a particular resource being rejected based on previous experience, and is often triggered by the resource’s odour, we introduce the broader term; “abandonment”. Abandonment refers to a reduction in the overall ant presence within the area where the danger was located, while being neutral about the mechanism involved. It is likely that some sort of learning process is involved, wherein food-related cues are associated with harm in some way—potentially via malaise and a subsequent avoidance of the most recent feeding area. In addition, it seems likely that some sort of communication is involved, amplifying avoidance beyond only the ants which directly fed on the bait. Ants might modulate activity on the trail towards a harmful food through the use of pheromones, for example by employing a negative chemical mark^86^ or by varying the balance of different pheromones to locally discourage exploration^87^. Again, however, the mechanisms behind this are not clear. Further investigation of the underlying mechanisms of this complex and highly adaptive behaviour will be critical to fully understanding, and perhaps, overcome it.

The rapid active abandonment of toxic baits we observed has large implications for ant control, and for toxicological research. In terms of concrete implications for control or eradication programs, maximizing bait consumption within the first two hours of discovery is key. It is essential to maximize the toxicant’s entry into the nests within the narrow timeframe between bait placement and ants recognizing its effects. This may be achieved by placing baits densely across an area and using bait stations that ensure unlimited simultaneous access for the ants. Using behaviour-modulating molecules to manipulate decision-making and enhance recruitment or quicker return to the feeder could be beneficial. For instance, synthetic pheromones added to bait stations may be helpful^88^; this has also been shown to be effective when added to toxic sprays^46,89^. The use of substances such as ketanserin, which increases toxic bait consumption^90^, or caffeine, which improves memory formation^91^, have been explored and may be helpful in improving bait uptake. Modifications to deployment protocols could also manipulate ant behaviour. Field and lab studies have revealed that an alternative two-step protocol with added odour enhances bait acceptance and ingestion of toxic baits^71^. Two-step protocols might also involve altering the characteristics and location of the baits after the initial 3 hours, so that they represent a new source for the ants rather than the previously abandoned one.

In terms of toxicological research, our results imply that palatability assays should only assess immediate responses to baits, or quantify consumption within the first two hours. Past this point, palatability may be masked by abandonment. This may in fact be good news: toxic bait formulations previously determined to be unpalatable on the basis of long-term consumption data may in fact not be unpalatable, but only seem to be so due to an abandonment effect^92^. Finally, our results have large implications for assessing the efficacy of control and eradication attempts. The field efficacy of toxic baits is usually measured by monitoring bait stations located in proximity to the toxic baits^41,68,93,94^. A decrease in ant activity at these monitoring stations is interpreted as being due to a mortality-based reduction in the population^69^. When ants reappear after a certain period, it is usually assumed that there was a reinvasion from the periphery^93,95,96^. However, it is not yet clear how long the abandonment effect lasts. Thus, a reduction of consumption at monitoring stations located close to the baits or a decrease in ant activity in the baited area may not be due entirely to mortality, but rather a combination of mortality and active abandonment. Unfortunately, even search and scan sampling may not be effective if the ants remain in the nest due to the abandonment behaviour. In field control situations, simultaneous toxic baits are numerous and broadly distributed. Ants may well avoid foraging in the whole area, or remain in their nests while the risk persists. For example, when parasitoid phorid flies are present, ants may remain underground^97–99^, and reduce foraging^100,101^ in response.

Our study demonstrated rapid active toxic bait abandonment by invasive ants. This has large implications for control efforts, bait assessment, and monitoring. Gaining a deeper understanding of the mechanisms that drive this collective response will provide insights into the mechanisms and strategies of behavioural immunity that social insects can deploy. Perhaps more crucially, a deeper understanding of these processes will be crucial for effectively addressing this complex behaviour. Recognizing that ants may remain present but evade baits until the perceived risk has subsided, we can implement modifications in order to boost toxicant entry into the nest, thereby improving efficacy. Social insects possess intricate and effective behavioural protection mechanisms. Understanding these will be a key step in grasping their social organization, and in controlling them when necessary.

## Methods

### Sampling area and times

The experiments were carried out on the campus of the University of Buenos Aires. This area is heavily infested by *Linepithema humile*, with many active trunk trails. The experiments were conducted during the warmer months of December – April 2021, 2022 and 2023, when ants are most active and many trunk trails can be located around the perimeter of buildings.

### Solution and toxicant

A 20% (w/w) sucrose solution was used, as this is well accepted by ants and is the most commonly used bait for this species^35,93,102^. The toxic bait was prepared by adding 3% w/w boric acid (Biopack) to the sucrose solution. Boric acid was chosen because Argentine ants respond well to this bait^54,103^. Additionally, it causes delayed mortality, which is critical for effective control^55^. The solutions were made with table sugar and tap water. We employed a relatively high concentration of boric acid, exceeding the recommended levels^92^. This decision was based on preliminary tests that demonstrated its acceptance on trunk trails. Additionally, we aimed to expedite the abandonment dynamics.

### Palatability test

First, we needed to confirm that the toxic bait to use in the experiments is palatable to this ant species under field conditions, to differentiate a potential abandonment effect from unpalatability. Previous laboratory studies on individual ants under controlled conditions showed that boric acid baits are palatable for the Argentine ants^54,103^. The palatability test evaluates the immediate response of the ant upon encountering the bait. Responses to toxic bait (20% sucrose + 3% boric acid), 20% sucrose solution, and, in order to visualize effective rejections a negative control of 20% sucrose and saturated quinine (c. 0.26 g/L) solution were evaluated. For that, a 1cm^2^ piece of plastic sheet with a drop of the solution to be tested was placed next to a highly active *L. humile* trunk trail. The response of the ants that touched the drop with their antennae or mouthparts was recorded for a minute. If they remained drinking (mandibles in contact with solution) for more than 4 consecutive seconds after contacting the drop, this was considered an acceptance. By contrast, if the ant quickly withdrew, this was considered a rejection^71^. This methodology enables us to promptly assess the instant and spontaneous responses of ants in the field without disturbing their trails.

### Experiment overview and general experimental design

We ran two field experiments (1 and 2) in this study:

1. Day-wise dynamics and spatial extent of trail abandonment
2. Hour-wise dynamics of trail abandonment

**Fig. 5:**
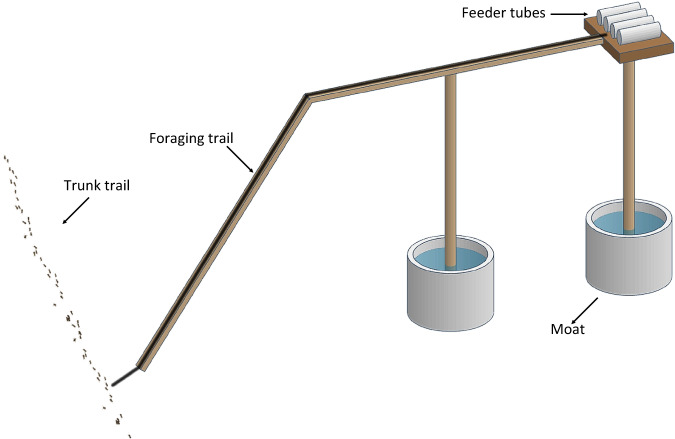
Experimental setup. Depiction of one of two identical bridges linked to a common trunk trail, spaced at least 7 meters apart (ants not to scale). Initially, both bridges offered sucrose solutions, allowing the formation of new foraging trails, with one later replaced to provide toxic bait.

Both experiments involve first offering trunk trails of the ant *L. humile* an unadulterated sucrose solution via a bridge to a set of feeders. This resulted in a new foraging trail leading from the trunk trail to the feeders. After some time (depending on the experiment) feeders were replaced with a palatable toxic bait (sucrose solution containing boric acid). We define *Trunk trails* as long paths with high ant activity which tend to persist over months or even years. We define *Foraging trails* as newly established paths that branch off from trunk trails towards a food source we provide (bridges; see Fig. 5)^104^. Only trunk trails at least 12 m long and with bi-directional traffic of more than 100 ants / minute, measured by counting ants crossing a fixed point on the trail, were used in the experiments.

Foraging trails were established by providing a food source (20% w/w sucrose in 4 cotton-plugged 9 ml tubes, henceforth: feeder tubes) on a foraging platform (8 × 5.5 cm) at the end of a bridge (Fig. 5). The bridges were made using light wooden slats (300 mm long, 10 mm wide, 5 mm thick). Each bridge was composed of two slats (30 cm each, 60 in total), one horizontal, and one articulated, angling downwards to allow access from the trunk trail. The bridge was raised on vertical posts (c. 25 cm high) surrounded by a water-and-detergent moat, to ensure that access to the platform was exclusively via the bridge entrance. The bridge was covered with painters’ tape which was replaced when starting each experiment and replicate.

Bridges were positioned in pre-established locations, contacting a point on a trunk trail so that the bridge entrance abutted an active trunk trail (Fig. 5). Feeder tubes were renewed at the beginning and at the end of each day of the experiments. Nonetheless, occasionally in the morning the sucrose tubes were empty.

#### Initiation of foraging

To stimulate the ants to climb the bridge and begin foraging, a plastic sheet (2×7 cm) with drops of 20% w/w sucrose was placed beside the trunk trail, directly beside the bridge entrance. Once filled with foraging ants, the sheets were carefully placed on the foraging arena. This caused the ants, after feeding, to descend from the bridge through the ramp, depositing pheromone and initiating a foraging trail on the bridges. During this phase, the feeder tubes were blocked.

#### Data collection

the main variable measured was *ant activity*, defined as the average number of ants crossing the measurement point over one minute. For the bridges, we counted the ants toward the foraging arena over 3 min, and on the trunk trail we counted ants in both directions for a minute. Before every activity measurement, we also recorded substrate temperature using a laser thermometer.

**Fig. 6:**
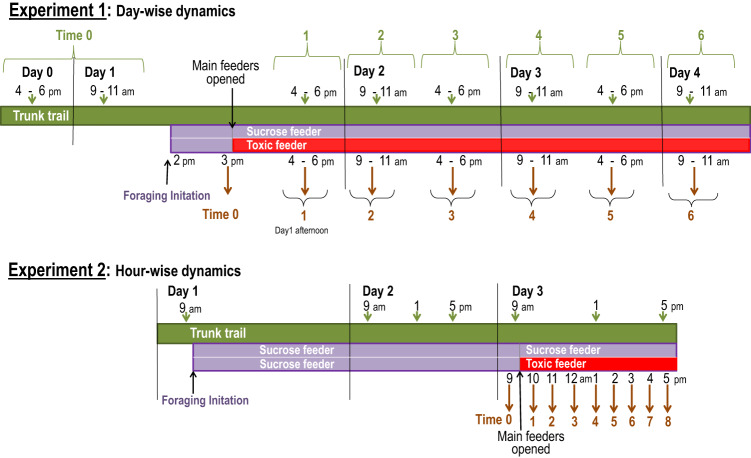
Timeline of Experiments 1 (day-wise dynamics) and 2 (hour-wise dynamics). The green bar represents the trunk trail, purple and red bars represent the bridges; purple when offering sucrose and red when offering toxic bait. In both experiments, initial activity was established by placing drops of sucrose solution on the foraging arena of both bridges while the tubes (main feeders) were blocked (thus both bridges offer plain sucrose, in purple). Braces and arrows indicate the time of ant activity measurements. This variable is, for the bridges, the average number of ants per minute crossing an imaginary line halfway across the bridge in the direction to the foraging arena, and for the trunk trail, in both directions. Time 0 represent the baseline just before opening the main feeders, i.e., when both bridges offered sucrose solution; at 9 am for the foraging trails (bridges), and the average of measurements of day 0 and morning day 1 for the trunk trail. Similarly, time 1, time 2, etc. represent the times when the ant activity was measured on the trunk trail, and at the foraging trails, after toxic bait became available. For Experiment 1, each time is an average of counts made over a ~2-h period, thus each time represents a morning or an afternoon, over four days. For Experiment 2 (hour-wise dynamics), the activity was measured hourly over a single day, each time representing an hour.

To demonstrate that any observed reduction in ant activity on the bridge was not caused by sugar satiation, we simultaneous offered a second bridge and feeder setup (Sucrose bridge) on the same trunk as the treatment setup (Toxicant bridge), with identical sham manipulations, offering unadulterated sucrose solution. For an overview of the experimental procedure, see Fig. 6.

To determine if trail abandonment is determined by population decline, activity on the trunk trail was measured at the two points where the bridges contacted it (and at other points in Experiment 1).

### Experiment 1: Day-wise dynamics and spatial extent of trail abandonment

The aim of this experiment was to experimentally demonstrate that ants respond to the toxic bait presence by abandoning the foraging trail, to describe spatial scale of this abandonment, and its temporal dynamics at a time scale of days.

This experiment began in the afternoon, to coincide with the time of increasing foraging activity (R. Josens and D. Zanola, pers. obs.). Two points on a trunk trail separated from each other by at least 7 meters were chosen, and a bridge was placed at each point. During the foraging initiation phase, activity on both bridges was measured. Adjusting the amount of sucrose solution offered allowed us to balance foraging activity on both bridges, except in one highly active replicate, where equalizing activity in both bridges was not possible.

One hour later (at 3 pm), the initial activity or *baseline* (time 0) on both bridges was recorded. Immediately after this measurement the main feeder tubes were unblocked. One bridge offered unadulterated sucrose (sucrose bridge), the other toxicant-laced sucrose (toxicant bridge). An hour later, three measurements were taken over a ~2-h period, (in fact 125 min) at one-hour intervals (4, 5, and 6 p.m.) to obtain the ant activity of this afternoon by averaging these values. Over the next two days, we recorded activity over 2-h periods in the morning (9–11 a.m.) and in the afternoon (4–6 p.m.). This provided a mean activity measurement for afternoon day 1, morning day 2, afternoon day 2, etc. (times 1, 2, 3, etc. in Fig. 6).

To determine if abandonment is limited to the foraging trail or if it also affects overall trunk trail activity, we also measured trunk trail activity during this experiment at different distances around the toxicant bridge (every meter up to 4 meters on either side). This allowed us to determine the spatial extent of the foraging decline and also to characterize its temporal dynamics.

Measurements on the trunk trail were also taken one day before placing the bridges (afternoon, day 0) and in the morning before placing the bridges (morning, day 1). The average of these measurements constitutes the *baseline activity* for the trunk trail (Time 0 in Fig. 2). Trunk trail activity was then measured at the same times as the foraging trail measurements (Times 1, 2, 3, etc. See Experiment 1 in Fig. 2).

This experiment was replicated 5 times on 5 different trunk trails.

### Experiment 2: Hour-wise dynamics of trail abandonment

Results from experiment 1 indicated that trail abandonment occurs rapidly, within 18 h of access to toxic bait. The aim of experiment 2 was to define the dynamics of abandonment at a higher temporal resolution. The methods were identical to experiment 1, with two differences. Firstly, after the activity initiation phase there was a *foraging increase phase* of two days, in order to generate a higher initial activity by offering sucrose solutions in both bridges over a longer time. Secondly, activity measurements were taken every hour, the baseline at 9 am with both bridges offering sucrose solutions, changing the feeder tubes immediately thereafter: plain sucrose on the *sucrose bridge*, and toxicant-laced sucrose in the *toxicant bridge*. One hour later, measurements began, at 10 am (time 1), and continued hourly until 5 pm (time 8) (see Fig. 2, Experiment 2). In light of the results of the trunk path in experiment 1, measurements of trunk trail activity were only performed at 2 places and only at 3 times: Trunk trail activity was recorded at 9 am, 1 pm, and 5 pm (day 2 and day 3) (Fig. 2). Activity on the trunk trail was measured adjacent to the bridges´ locations.

This experiment was replicated 6 times on 6 different trunk trails.

### Assessing mortality

Finally, in order to assess whether the reduction observed in the field could have been due to rapid mortality caused by ingestion of the toxic bait, we conducted a laboratory test. We evaluate individual ants from three laboratory nests of *L. humile* after depriving them of carbohydrates for 48 h. We placed 5 worker ants in 5 cm diameter plastic containers with fluon-coated walls. Half of the groups received a droplet of sugar solution on a small plastic sheet, while the other half received the boric acid bait, prepared in the same manner as in the field trials. We left the drop for 10 minutes, verifying that all ants ingested the solution. Then, the sheet with the drop was carefully removed. From there, we let an hour pass and then counted the number of dead ants every hour for 6 hours.

### Statistics and reproducibility

For experiments 1 and 2, we recorded the activity of ants at different time points: 1) mornings and afternoons over several days and 2) hourly on the foraging trails (bridges) and at various locations along the trunk trail. We focused only on a few key time points and locations along the trunk trail for statistical analyses (repeated measures design). We used GLMMs for the analysis of all experiments data. In all cases the response variable was Ant activity, measured as the average ants crossing a line per minute. For the bridges, only traffic in the direction of the foraging arena was counted; for the trunk trail both directions were included. Homoscedasticity assumption was assessed using a standardized residuals vs predicted values plot (Supplementary Fig. S1, S5, S7, S9, S13). The best-fitting distribution for the data was determined by the dispersion index (the ratio between the residuals and the predicted variance), which was found to be the negative binomial distribution in all cases. Pairwise comparisons of activity were conducted using the emmeans package^105^ (Supplementary Fig. S2, S3, S6, S8, S10, S11, S14). P value adjustment was applied by using dunnettx method. Statistical analyses were performed in R Studio using the glmmTMB, nlme, and multcomp packages^106–108^.

A detailed description of the statistical analysis approach taken and the entire code and statistical output are provided in the Supplementary Material. In short, for all analyses we compare the number of ants on sucrose and toxicant bridges at various time points after provision of the toxic bait to the number of ants present just before toxic bait provision (which we consider the baselines).

### Reporting summary

Further information on research design is available in the Nature Portfolio Reporting Summary linked to this article.

### Supplementary information

Supplementary Information

Reporting Summary

## Data Availability

The data supporting the findings of this study are available from https://figshare.com/s/18abcfa3e89b5dd68d00^109^.
