# Supplementary material for: Ants evade harmful food by active abandonment
Source: Commun Biol. 2024 Jan 12;7:84. doi: 10.1038/s42003-023-05729-7 (PMC10786876; doi:10.1038/s42003-023-05729-7)
Supplement: Supplementary file 1 — Supplementary Information [file 42003_2023_5729_MOESM1_ESM.pdf]

Supplementary information for

Behavioural immunity: ants evade harmful food by active abandonment

Daniel Zanola[1], Tomer J. Czaczkes[2] and Roxana Josens[1]*

[1] Laboratorio de Insectos Sociales, Departamento de Biodiversidad y Biología Experimental, Facultad de Ciencias Exactas y Naturales, Universidad de Buenos Aires, IFIBYNE, CONICET, Ciudad Universitaria Pab. II. (C1428 EHA), Buenos Aires, Argentina

[2] Animal Comparative Economics laboratory, Faculty of Biology and Preclinical Medicine, University of Regensburg, Universitaetsstrasse 31, 93053 Regensburg, Germany

* Corresponding author: Roxana Josens.

Email: roxy@bg.fcen.uba.ar

Table of contents

Statistical Analyses
I. General explanation for the statistical analyses 2
II. Foraging Trail (bridges) Dynamics Over Days 2
Details of the Model for the bridges over days: 3
Contrasts between treatment B-S 5
Contrasts t0 vs. each of the times per treatment 6
Model for only time 1 in detail for the bridges: 8
Details of the Model only t1 for bridges 9
Contrast t0 vs each of the times per treatment 11
III. Trunk Trail Dynamics Over Days 13
Details of the Model for trunk trail over days 13
IV. Foraging Trail (bridges) Dynamics Over Hours Throughout a Day 19
Details of the Model for the bridges over hours 19
Contrasts Baseline (9hs) vs. each of the times per treatment 21
Contrasts between treatment B-S 23
V. Trunk Trail Dynamics Over Hours Throughout a Day 26
Results: 26
Details of the Model for trunk trail over hours 27
Contrasts Baseline (9hs) vs. each of the times per treatment 29
VI. Assessing Mortality 31
VII. Supplementary References 31

STATISTICAL ANALYSES

I. General explanation for the statistical analyses

In all cases the response variable was *Ant activity* measured as the average number of ants crossing a line over one minute. For the bridges, only traffic in the direction of the foraging arena was counted; for the trunk trail both directions were included.

Statistical analyses were performed in R Studio using the glmmTMB, nlme, and multcomp packages (Brooks et al., 2023; Hothorn et al., 2023; Pinheiro et al., 2023). Homoscedasticity assumption was assessed using a standardized residuals vs predicted values plot using the Dharma (Hartig, 2020). Alternative probability distributions were explored to find the best fit for the data and/or variance using the Akaike information criterion (AIC). Pairwise comparisons of activity were conducted using the emmeans package (Searle et al., 1980) and effect sizes were assessed when significant differences were found.

The entire code and statistical output are provided as in the following sections.

II. Foraging Trail (bridges) Dynamics Over Days

For the foraging trails (bridges), descriptive analyses were conducted using ggplot2 (Wickham, 2014) to assess the data dynamics over time. Subsequently, a statistical analysis was performed using a linear mixed-effects model. The response variable was ant activity on each bridge (mean number of ants per minute), and the distribution that best fit the data was the negative binomial distribution. The fixed explanatory variables included the treatments (2 levels: toxicant and control bridges) and time points (7 levels: time 0 (Day 1: 15:00); time 1 (Day 1: 16:00-18:05); time 2 (Day 2: 9:00-11:00); time 3 (Day 2: 16:00-18:00); time 4 (Day 3: 9:00-11:00); time 5 (Day 3: 16:00-18:00); and time 6 (Day 4: 9:00-11:05)). The random explanatory variables were the replicates (n = 5) and the bridges (10 levels: two bridges per replicate). This resulted in the following model formula:

Ant activity = Treatment * Time + (random effects: bridge nested in Replicate), distribution family: negative binomial (log link function).

Lastly, pairwise comparisons of activity between treatments were performed using the emmeans:contrast() function.

Details of the Model for the bridges over days:

Each morning is a time and each afternoon is a time (time also named “t” in some analyses. Each one of these times is the average of 3 mean values recorded in a 125 min period (for example: 3 min at 16hs, 3 min at 17hs and 3 min at 18hs. Each one expressed per minute).

Variable response: Activity (mean count of ants/min)

Treatments: (Bridge Sucrose and Bait, S and B respectively. 2 levels)

Time (factor): t0 (Day 1: 15hs) – t1 (Day 1: 16 to 18hs) – t2 (Day 2: 9 to 11hs) – t3 (Day 2: 16 to 18hs) – t4 (Day 3: 9 to 11hs) – t5 (Day 3: 16 to 18hs) – t6 (Day 4: 9 to 11hs). *7 levels*

Bridges: Random Variable. 10 levels

Replicas: n: 5

simres <- simulateResiduals(model_day_bridges) # check the model fits using DHARMa package

plot(simres)

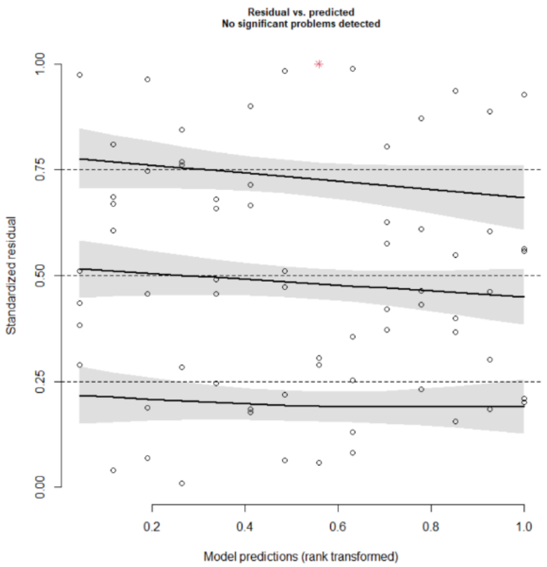

Figure S1. Residuals vs. Predicted to validate model_day_bridges using the Dharma.

model_day_bridges

```
model_day_bridges <- glmmTMB(Activity ~ Treatment*Time + (1 | Replica/Bridge), data = day_bridges, family = nbinom1(link = "log"))

summary(model_day_bridges)
```

Set of variables considered in the generalized linear (mixed) model day bridges:

```
Family: nbinom1 ( log )
Formula:          Activity  ~ Treatment * Time + (1 | Replica/Bridge)
Data: day_bridges

     AIC      BIC   logLik deviance df.resid
   618.6    656.8   -292.3    584.6       53

Random effects:

Conditional model:
 Groups         Name        Variance Std.Dev.
Bridge:Replica (Intercept) 0.01361  0.1167
 Replica        (Intercept) 0.18567  0.4309
Number of obs: 70, groups:  Bridge:Replica, 10; Replica, 5

Dispersion parameter for nbinom1 family (): 7.75
```

Conditional model:

```
                     Estimate Std. Error z value Pr(>|z|)
(Intercept)           3.82293    0.27101  14.106  < 2e-16 ***
TreatmentS            0.06245    0.26407   0.236 0.813046
Timet1               -0.33597    0.27609  -1.217 0.223646
Timet2               -1.39978    0.38938  -3.595 0.000324 ***
Timet3               -2.23762    0.49539  -4.517 6.28e-06 ***
Timet4               -1.13835    0.34564  -3.293 0.000990 ***
Timet5               -1.50270    0.40563  -3.705 0.000212 ***
Timet6               -1.61995    0.41656  -3.889 0.000101 ***
TreatmentS:Timet1     0.56118    0.36274   1.547 0.121849
TreatmentS:Timet2     1.87637    0.45001   4.170 3.05e-05 ***
TreatmentS:Timet3     2.94392    0.54032   5.448 5.08e-08 ***
TreatmentS:Timet4     1.61126    0.41202   3.911 9.20e-05 ***
TreatmentS:Timet5     2.17162    0.46103   4.710 2.47e-06 ***
TreatmentS:Timet6     1.87533    0.47799   3.923 8.73e-05 ***
---
Signif. codes:  0 '***' 0.001 '**' 0.01 '*' 0.05 '.' 0.1 ' ' 1
```

```
sum (residuals(model_day_bridges,type ="pearson")^2)/df.residual(model_day_bridges)
[1] 1.202891
```

Contrasts between treatment B-S

```
***the emmeans1***
meanie1 <- emmeans(model_day_bridges, pairwise ~ Treatment | Time)
print (meanie1)
```

$contrasts

Values in bold represent p-values<0.05

Signif. codes: 0 ‘***’ 0.001 ‘**’ 0.01 ‘*’ 0.05 ’(*)’ 0.1 ‘NS’ 1

Time = t0:

contrast	estimate	SE	df	t.ratio	p.value
B - S	-0.0625	0.264	53	-0.236	0.8140

Time = t1:

contrast	estimate	SE	df	t.ratio	p.value
B - S	-0.6236	0.273	53	-2.280	**0.0266**

Time = t2:

contrast	estimate	SE	df	t.ratio	p.value
B - S	-1.9388	0.379	53	-5.120	**<.0001**

Time = t3:

contrast	estimate	SE	df	t.ratio	p.value
B - S	-3.0064	0.482	53	-6.241	**<.0001**

Time = t4:

contrast	estimate	SE	df	t.ratio	p.value
B - S	-1.6737	0.335	53	-4.997	**<.0001**

Time = t5:

contrast	estimate	SE	df	t.ratio	p.value
B - S	-2.2341	0.389	53	-5.736	**<.0001**

Time = t6:

contrast	estimate	SE	df	t.ratio	p.value
B - S	-1.9378	0.412	53	-4.706	**<.0001**

Results are given on the log (not the response) scale.

```
plot(meanie1)
```

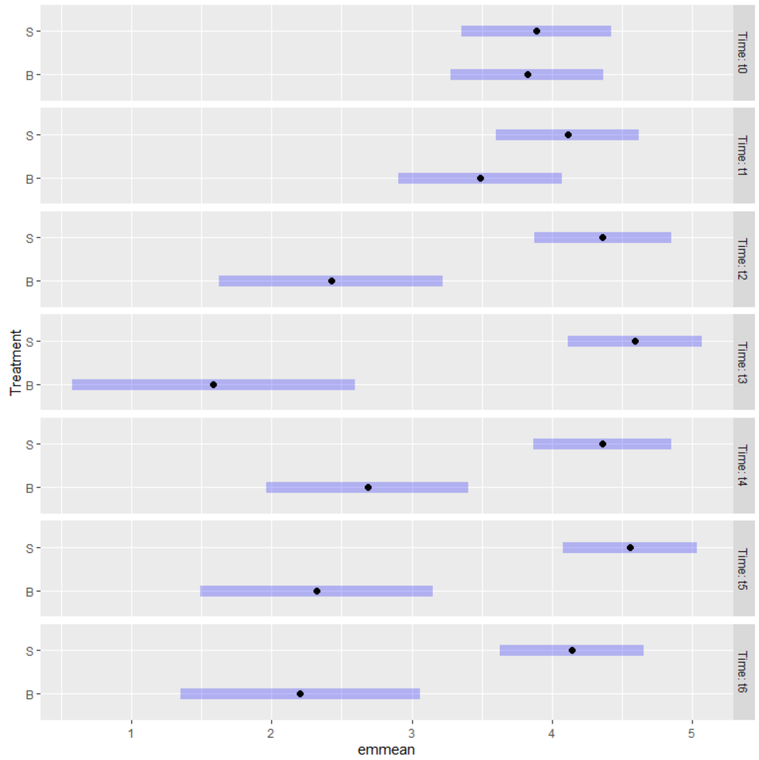

Figure S2. Emmean contrasts between treatments (S-B) at different times in model_day_bridges.

Contrasts t0 vs. each of the times per treatment

*** the emmeans2***

meanie2 <- emmeans(model_day_bridges, pairwise ~ Time | Treatment)

meanie.contrasts <- contrast(meanie, method = "trt.vs.ctrl", reverse = "true")

print (meanie.contrasts)

$emmeans

Values in bold represent p-values<0.05

Signif. codes: 0 ‘***’ 0.001 ‘**’ 0.01 ‘*’ 0.05 ’(*)’ 0.1 ‘NS’ 1

Treatment = B:

contrast	estimate	SE	df	t.ratio	p.value	
t0 - t1	0.336	0.276	53	1.217	0.6475	NS
t0 - t2	1.400	0.389	53	3.595	**0.0039**	**
t0 - t3	2.238	0.495	53	4.517	**0.0002**	***
t0 - t4	1.138	0.346	53	3.293	**0.0095**	**
t0 - t5	1.503	0.406	53	3.705	**0.0028**	**
t0 - t6	1.620	0.417	53	3.889	**0.0016**	**

Treatment = S:

contrast estimate SE df t.ratio p.value

t0 - t1	-0.225	0.235	53	-0.957	0.7986	NS
t0 - t2	-0.477	0.225	53	-2.114	0.1706	NS
t0 - t3	-0.706	0.218	53	-3.243	**0.0110**	*
t0 - t4	-0.473	0.227	53	-2.082	0.1817	NS
t0 - t5	-0.669	0.219	53	-3.052	**0.0186**	*
t0 - t6	-0.255	0.237	53	-1.076	0.7329	NS

Results are given on the log (not the response) scale.
P value adjustment: dunnettx method for 6 tests

tapply(Activity _**b**$Activity, Activity_b$Time, FUN = mean)

t0	t1	t2	t3	t4	t5	t6
50.6	34.8	12.4	6.0	13.4	12.6	9.8

tapply(Activity _**s**$ Activity, Activity _s$Time, FUN = mean)

t0	t1	t2	t3	t4	t5	t6
63.2	71.2	85.6	108.6	86.0	104.0	70.0

<u>%Change activity compared to initial (t0)</u>

Treatments = B:

t1 = 31.2 % smaller than the initial

t2= 75.5 % smaller than the initial

t3= 88.1% smaller than the initial

t4= 73.5% smaller than the initial

t5=75.1% smaller than the initial

t6=80.6% smaller than the initial

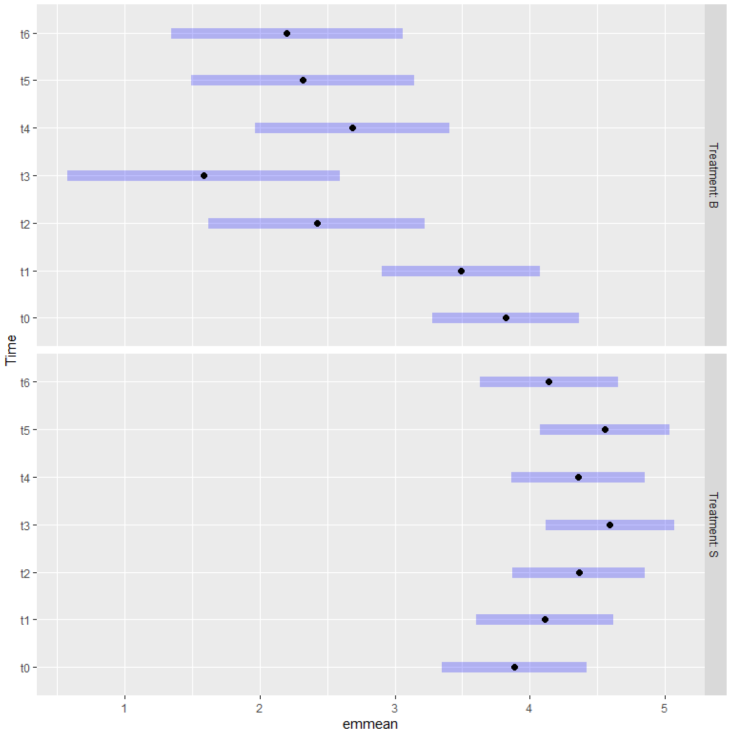

Figure S3. Emmean contrasts of the times within each treatment (B-S) in model_day_bridges.

Given the distinct behaviour observed at time 2 (1st morning after bait access) at the toxicant bridge compared to its baseline, and the resemblance between time 1 and the baseline, we intend to conduct a separate analysis for each of the three measurements within time 1 to evaluate if there in differences or tendencies appeared.

Model for only time 1 in detail for the bridges:

Only the first three hours after feeders opening are analysed here. So, each ***t*** is the mean count of ants/min at this hour. Instead of using the average of the 3 hours as in the previous analysis, here we used the mean activity of each hour within the first afternoon.

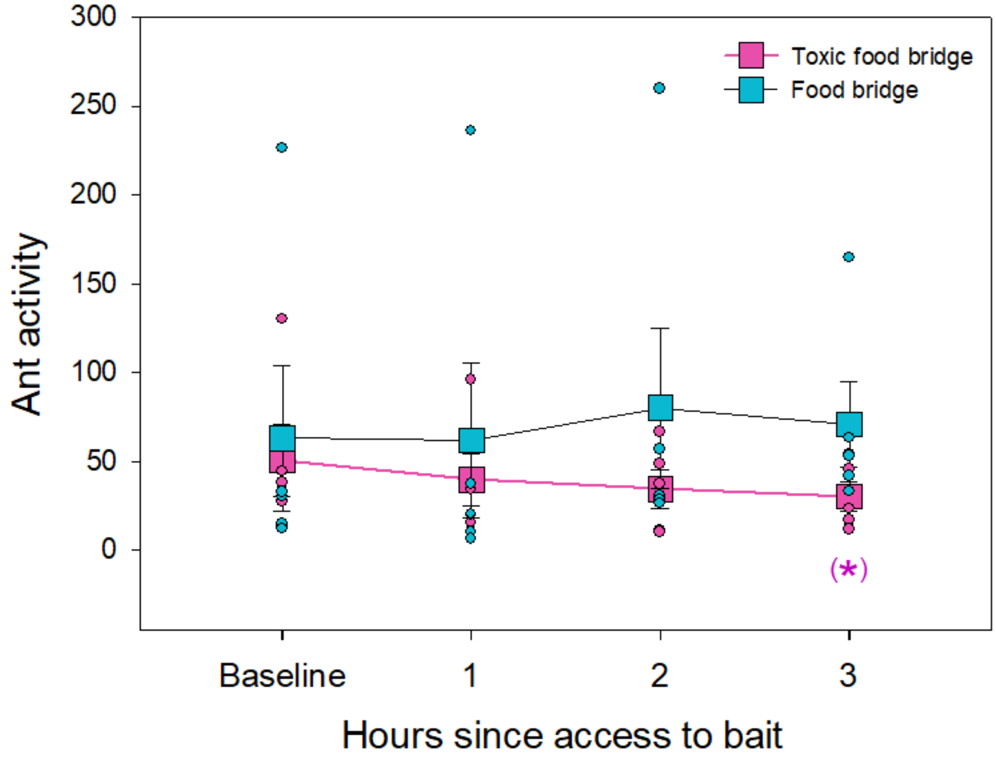

Figure S4: Detail of the first afternoon (time 1: 1, 2 and 3h after bait access) broken down into each of its 3 measurements. Pink for the bridge offering the toxic bait and light blue for the bridge offering plane sucrose solution. Circles are the data points and squares the mean +/- SE. (*): P = 0.0057.

Details of the Model only t1 for bridges

Response Variable: Activity (mean count of ants/min)

Treatments: (Bridge Sucrose and Bait, 2 levels)

Time (factor): 15hs (Baseline) – 16hs – 17hs – 18hs *(4 levels)*

Bridges: Random Variable. 10 levels

Replicas: n: 5

simres <- simulateResiduals(model_t1) # check the model fits using DHARMa package

plot(simres)

Figure S5. Residuals vs. Predicted to validate model_t1 using the Dharma.

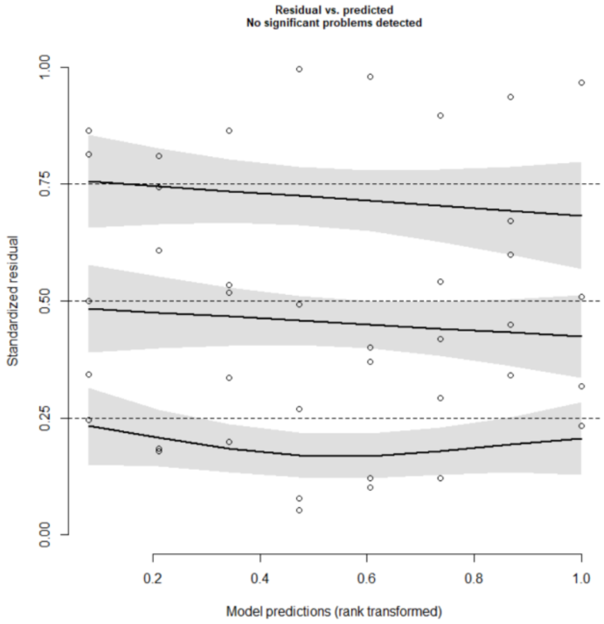

model_t1 = glmmTMB(Activity ~ Treatment * Hour + (1|Replica/Bridge), data = Data3, family = nbinom1(link = "log"))

summary(model_t1)

Set of variables considered in the generalized linear (mixed) model t1.

Family: nbinom1 (log)

Formula: Activity ~ Treatment * Hour + (1 | Replica/Bridge)

Data: Data3

AIC	BIC	logLik	deviance	df.resid
370.3	388.9	-174.2	348.3	29

Random effects:

Conditional model:

Groups	Name	Variance	Std.Dev.
Bridge:Replica	(Intercept)	0.08136	0.2852
Replica	(Intercept)	0.46719	0.6835

Number of obs: 40, groups: Bridge:Replica, 10; Replica, 5

Dispersion parameter for nbinom1 family (): 4.59

```
Conditional model:
                     Estimate Std. Error z value Pr(>|z|)
(Intercept)           3.74981    0.36429  10.293   <2e-16 ***
TreatmentS           -0.01738    0.27318  -0.064   0.9493
Hour16hs             -0.22076    0.21669  -1.019   0.3083
Hour17hs             -0.35629    0.22558  -1.579   0.1142
Hour18hs             -0.60896    0.25006  -2.435   0.0149 *
TreatmentS:Hour16hs   0.16915    0.28651   0.590   0.5549
TreatmentS:Hour17hs   0.60056    0.28528   2.105   0.0353 *
TreatmentS:Hour18hs   0.69984    0.31000   2.258   0.0240 *
---
Signif. codes:  0 '***' 0.001 '**' 0.01 '*' 0.05 '.' 0.1 ' ' 1
sum(residuals(model_t1,type ="pearson")^2)/df.residual(model_t1)
[1] 1.089469

meanie <- emmeans(model_t1, pairwise ~ Hour | Treatment)
meanie.contrasts <- contrast(meanie, method = "trt.vs.ctrl", reverse = "true")
print (meanie.contrasts)
```

Contrast t0 vs each of the times per treatment

$emmeans

Values in bold represent p-values<0.05

Signif. codes: 0 '***' 0.001 '**' 0.01 '*' 0.05 '(*)' 0.1 'NS' 1

Treatment = B:

contrast	estimate	SE	df	t.ratio	p.value	
15hs - 16hs	0.2208	0.217	29	1.019	0.6078	NS
15hs - 17hs	0.3563	0.226	29	1.579	0.2904	NS
15hs - 18hs	0.6090	0.250	29	2.435	0.0567	(*)

% Change activity with respect to the initial

18hs= 39.92% smaller than the initial (15hs)

Treatment = S:

contrast	estimate	SE	df	t.ratio	p.value	
15hs - 16hs	0.0516	0.187	29	0.276	0.9694	NS
15hs - 17hs	-0.2443	0.175	29	-1.399	0.3806	NS
15hs - 18hs	-0.0909	0.183	29	-0.498	0.8974	NS

Conclusion: From the 3rd hour onwards, there is a tendency to decrease the activity in the toxic bridge, while the activity remained similar in the sucrose bridge.

Results are given on the log (not the response) scale.

P value adjustment: dunnettx method for 3 tests

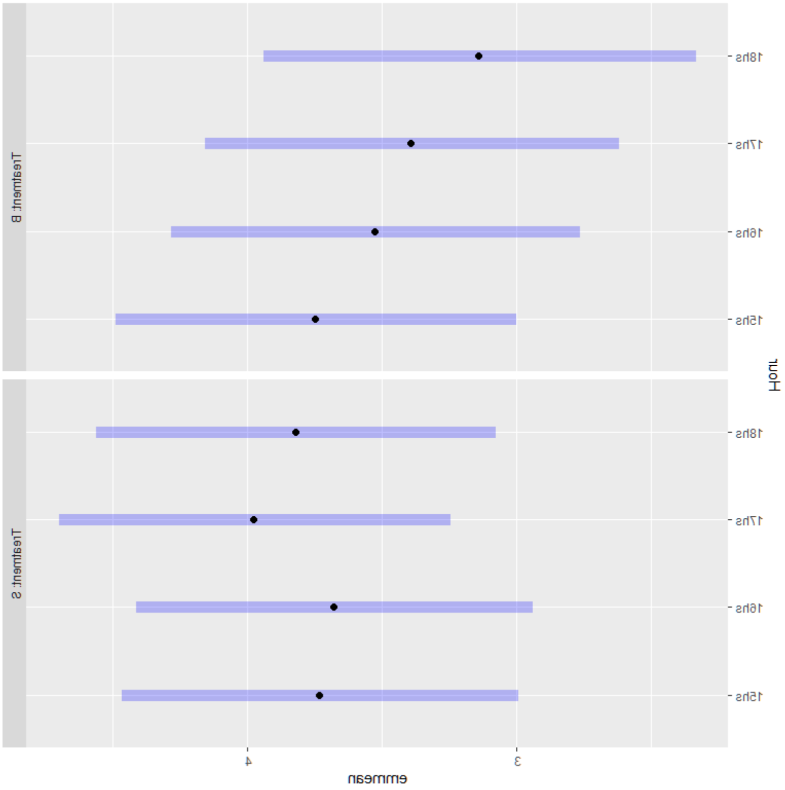

Figure S6. Emmean contrasts of the times within each treatment (B-S) in model_t1

III. Trunk Trail Dynamics Over Days

Here, we explored the spatial extent on the trunk trail of foraging abandonment. The response variable was the Ant activity (mean number of ants per minute) measured at different locations along the trunk trail.

We used a negative binomial distribution. The fixed explanatory variables included the qualitative variables for the locations (4 levels: 0m (toxicant bridge); 2m, 4m; 0mS (sucrose bridge)) and time points (7 levels: time 0 (afternoon day 0 and morning day 1), time 1 (afternoon day 1); time 2 (morning day 2); time 3 (afternoon day 2) up to time 6). The random explanatory variable was the replicates (n = 5). A linear mixed-effects model with interaction was performed. This resulted in the following model formula:

Ant activity = Sites * Time + (random effect: replicate), distribution family: negative binomial (log link function).

Finally, pairwise comparisons of activity between treatments were conducted using the emmeans:contrast function.

The time points of measurements coincided with those of the bridges, so it allows to evaluate the fine synchronization of the decrease in the activity between the trunk trail and Bait bridge. In this way, both have the same times.

So, let´s check if there was any variation in the activity on the trunk trail.

Details of the Model for trunk trail over days

Response Variable: Activity (mean count of ants/min)

Time: 0 (afternoon del day 0 and morning day 1) - 1 (afternoon day 1) – 2 (morning day 2) – 3 (afternoon day 2) until time 6 *(Resulting 7 levels)*

Sites: 0mB – 2m – 4m – 0mS (4 levels) (2m is the average from right and left; same for 4m)

Replica: n=5

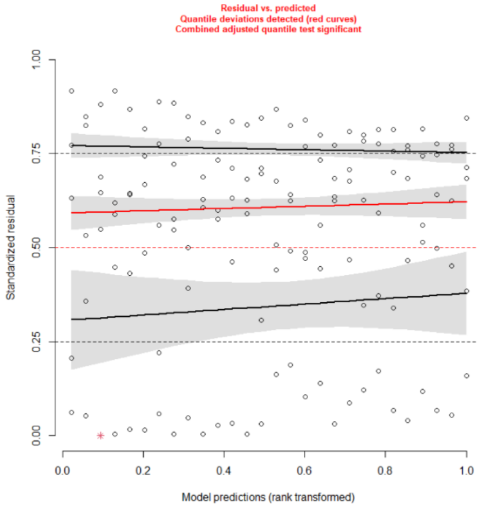

Figure S7. Residuals vs. Predicted to validate model_trunk_days using the Dharma.

model_trunk_days
model_trunk_days <- glmmTMB(Activity ~ Sites * Time + (1|Replica), data = trunk_days, family = nbinom1(link = "log"))

summary(model_trunk_days)

Set of variables considered in the generalized linear (mixed) model trunk day.

```
Family: nbinom1  ( log )
Formula:          Activity ~ Sites * Time + (1 | Replica)
Data: trunk_days
     AIC      BIC   logLik deviance df.resid
  1609.9   1698.2   -775.0   1549.9      110

Random effects:

Conditional model:
 Groups  Name        Variance Std.Dev.
 Replica (Intercept) 0.5773   0.7598
Number of obs: 140, groups:  Replica, 5

Dispersion parameter for nbinom1 family (): 12.9

Conditional model:
                          Estimate Std. Error z value Pr(>|z|)
(Intercept)               5.632433   0.351717  16.014  < 2e-16 ***
Sites0mS                  0.042384   0.124755   0.340 0.734054
Sites2m                   0.028536   0.125158   0.228 0.819644
Sites4m                   0.073388   0.123868   0.592 0.553533
Time1          -0.058409   0.127885  -0.457 0.647865
Time2          -0.117798   0.129857  -0.907 0.364336
Time3          -0.367892   0.139431  -2.639 0.008327 **
Time4          -0.344843   0.138306  -2.493 0.012655 *
Time5          -0.362418   0.138867  -2.610 0.009059 **
Time6          -0.565555   0.147602  -3.832 0.000127 ***
Sites0mS:Time1 -0.049448   0.180159  -0.274 0.783722
Sites2m:Time1  -0.085515   0.181549  -0.471 0.637619
Sites4m:Time1  -0.051257   0.178818  -0.287 0.774388
Sites0mS:Time2  0.245364   0.176641   1.389 0.164816
```

```
Sites2m:Time2  -0.092141   0.184534  -0.499 0.617558
Sites4m:Time2  -0.000672   0.180412  -0.004 0.997028
Sites0mS:Time3  0.447404   0.184699   2.422 0.015421 *
Sites2m:Time3   0.026380   0.194777   0.135 0.892268
Sites4m:Time3   0.158987   0.189374   0.840 0.401168
Sites0mS:Time4  0.374340   0.184781   2.026 0.042779 *
Sites2m:Time4   0.019250   0.193498   0.099 0.920755
Sites4m:Time4   0.083606   0.189856   0.440 0.659672
Sites0mS:Time5  0.397595   0.185121   2.148 0.031733 *
Sites2m:Time5  -0.068881   0.197029  -0.350 0.726639
Sites4m:Time5   0.001094   0.193072   0.006 0.995481
Sites0mS:Time6  0.422866   0.195386   2.164 0.030445 *
Sites2m:Time6  -0.003626   0.207251  -0.017 0.986042
Sites4m:Time6   0.139391   0.201003   0.693 0.488011
---
Signif. codes:  0 ‘***’ 0.001 ‘**’ 0.01 ‘*’ 0.05 ‘.’ 0.1 ‘ ’ 1
> sum(residuals(model_trunk_days,type ="pearson")^2)/df.residual(model_trunk_days)
[1] 0.9524291
```

the emmeans

```
meanie <- emmeans(model_trunk_days, pairwise ~ Time | Sites)

meanie.contrasts <- contrast(meanie, method = "trt.vs.ctrl", reverse = "true")

print (meanie.contrasts)
```

$emmeans

Values in bold represent p-values<0.05

Signif. codes: 0 ‘***’ 0.001 ‘**’ 0.01 ‘*’ 0.05 ’(*)’ 0.1 ‘NS’ 1

Sites = 0mB:

contrast	estimate	SE	df	t.ratio	p.value	
0 - 1	0.0584	0.128	110	0.457	0.9732	NS
0 - 2	0.1178	0.130	110	0.907	0.8234	NS
0 - 3	0.3679	0.139	110	2.639	**0.0479**	*
0 - 4	0.3448	0.138	110	2.493	0.0691	(*)
0 - 5	0.3624	0.139	110	2.610	0.0516	(*)
0 - 6	0.5656	0.148	110	3.832	**0.0012**	**

Sites = 2m:

contrast estimate   SE df t.ratio p.value

0 - 1	0.1439	0.129	110	1.116	0.7076	NS
0 - 2	0.2099	0.131	110	1.600	0.4040	NS
0 - 3	0.3415	0.136	110	2.503	0.0674	(*)
0 - 4	0.3256	0.136	110	2.402	0.0861	(*)
0 - 5	0.4313	0.140	110	3.081	**0.0140**	*
0 - 6	0.5692	0.146	110	3.906	**0.0009**	***

Sites = 4m:

contrast	estimate	SE	df	t.ratio	p.value	
0 - 1	0.1097	0.125	110	0.877	0.8381	NS
0 - 2	0.1185	0.125	110	0.945	0.8041	NS
0 - 3	0.2089	0.128	110	1.626	0.3890	NS
0 - 4	0.2612	0.130	110	2.005	0.2024	NS
0 - 5	0.3613	0.134	110	2.688	**0.0421**	*
0 - 6	0.4262	0.137	110	3.118	**0.0125**	*

Sites = 0mS:

contrast	estimate	SE	df	t.ratio	p.value	
0 - 1	0.1079	0.127	110	0.850	0.8508	NS
0 - 2	-0.1276	0.120	110	-1.065	0.7378	NS
0 - 3	-0.0795	0.121	110	-0.657	0.9253	NS
0 - 4	-0.0295	0.123	110	-0.241	0.9959	NS
0 - 5	-0.0352	0.122	110	-0.287	0.9930	NS
0 - 6	0.1427	0.128	110	1.115	0.7086	NS

Results are given on the log (not the response) scale.

P value adjustment: dunnettx method for 6 tests

```
tapply(b0$Activity, b0$Time, FUN = mean)
    0     1     2     3     4     5     6
344.2 321.2 302.2 242.8 247.8 236.2 197.0

tapply(m2$Activity, m2$Time, FUN = mean)
    0      1      2      3      4     5     6
353.2  304.20 280.80 255.00 254.60 231.2  201.6

tapply(m4$Activity, m4$Tieme, FUN = mean)
    0     1     2     3     4     5     6
371.6 334.8 327.4 300.8 284.0 267.2 245.0

tapply(s0$Activity, s0$Time, FUN = mean)
    0     1     2     3     4     5     6
355.4 324.4 405.6 385.4 365.2 368.0 308.2
```

<u>% Change activity with respect to the initial</u>

Site = 0mB:

t3 = 29.5 % smaller than the initial

t4 = 28 % smaller than the initial

t5= 31.4% smaller than the initial

t6= 42.8% smaller than the initial

Site = 2m:

t5= 34.5% smaller than the initial

t6= 42.9% smaller than the initial

Site = 4m:

t5= 28.1% smaller than the initial

t6= 34.1% smaller than the initial

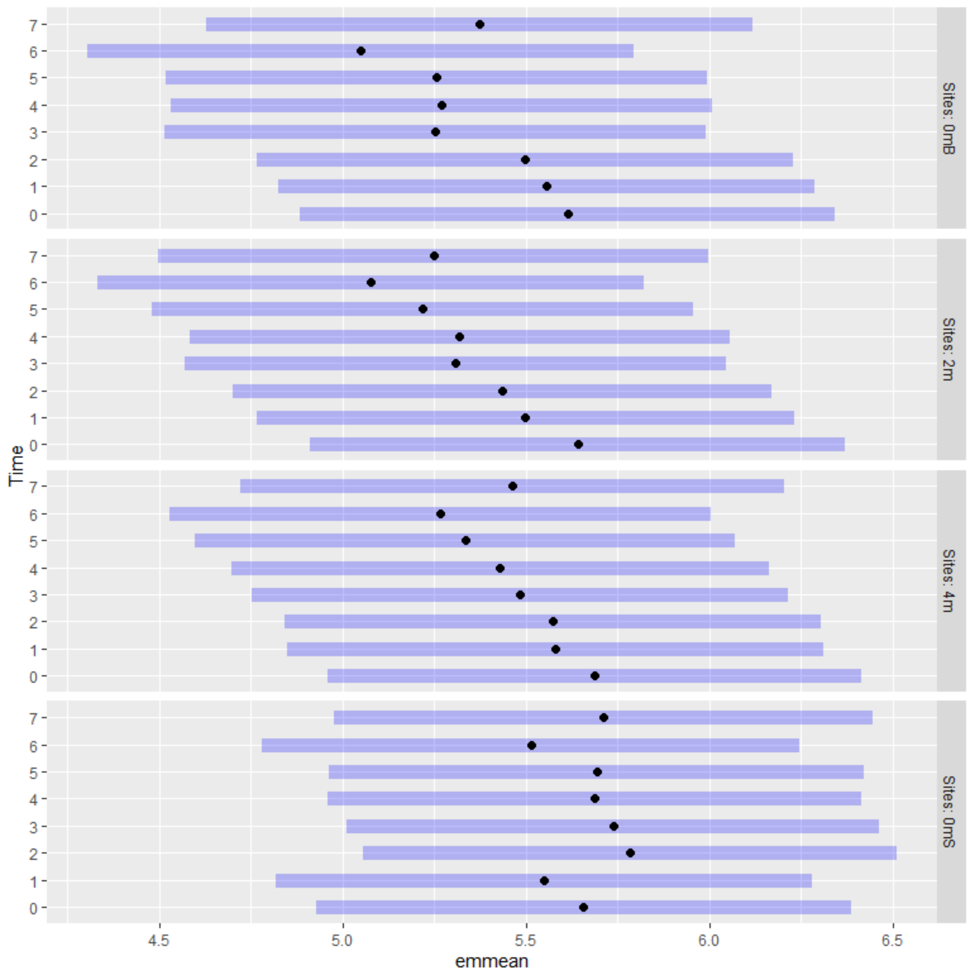

Figure S8. Emmean contrasts of the times within each site in model_trunk_days.

IV. Foraging Trail (bridges) Dynamics Over Hours Throughout a Day

This experiment consisted of 6 replicates. However, in one of the replicates, the temperature exceeded 40 degrees (°C) for a single recording at 2 p.m. As a result, there were no ants on the bridges during that specific time. However, the temperature dropped a few degrees by the next recording, and activity resumed at previous levels. Therefore, the data from both bridges for that particular replicate and only during that time point were excluded from the analysis.

The response variable was ant activity measured as the mean number of ants per minute on the bridges, and the best-fitting distribution for the data was the negative binomial distribution. The fixed explanatory variables included the treatments (2 levels: toxicant bridge and sucrose bridge) and time (9 levels: hourly from 9 am to 5 pm). Note that we chose to consider time as a factor, rather than a continuous variable, in order to facilitate pairwise comparisons. This was important to us, as we wanted to specify the timepoint at which activity began differing from baseline. The random explanatory variables were the replicates (n=6) and the bridges (12 levels: two bridges per replicate).

Activity = Treatment * Hour + (random effects: bridge nested in Replicate), distribution family: negative binomial (log link function).

Pairwise comparisons of activity between treatments were conducted using the emmeans:contrast() function.

Details of the Model for the bridges over hours

Response Variable: Activity (mean count of ants/min)

Treatments: (Bridge Sucrose and Bait, 2 levels)

Time (hours): from 9 to 17hs *(9 levels)* 9hs is the baseline

Bridges: Random Variable. 12 levels

Replicas: n: 6

Figure S9. Residuals vs. Predicted to validate model_hours_bridges using the Dharma.

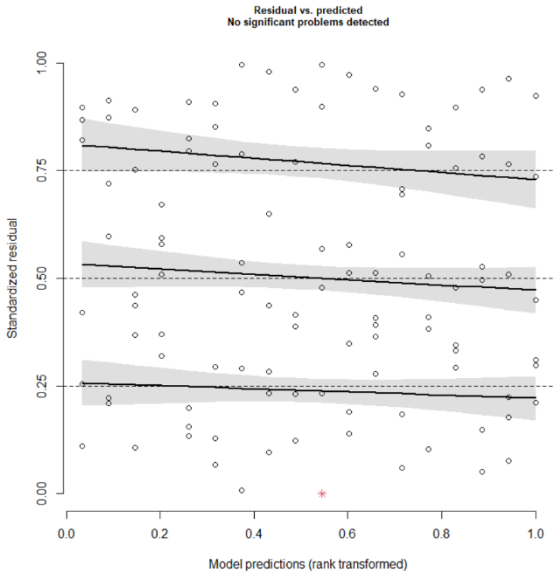

*** model_hours_bridges ***
model_hours_bridges = glmmTMB(Activity ~ Treatment * Hour + (1|Replica/Bridge), data = bridges_hour_n106, family= nbinom1(link = "log"))

summary(model_hours_bridges)

Set of variables considered in the generalized linear (mixed) model hours bridge.

Family: nbinom1 (log)

Formula: Activity ~ Treatment * Hour + (1 | Replica/Bridge)

Data: bridges_hour_n106

AIC	BIC	logLik	deviance	df.resid
1102.1	1158.0	-530.0	1060.1	85

Random effects:

Conditional model:

Groups	Name	Variance	Std.Dev.
Bridge:Replica	(Intercept)	0.005712	0.07558
Replica	(Intercept)	0.127580	0.35718

Number of obs: 106, groups: Bridge:Replica, 12; Replica, 6

Dispersion parameter for nbinom1 family (): 9.57

Conditional model:

	Estimate	Std. Error	z value	Pr(>|z|)	
(Intercept)	4.977714	0.183201	27.171	< 2e-16	***
Treatment-S	-0.222048	0.163665	-1.357	0.17487	
Hour10hs	0.001843	0.148797	0.012	0.99012	
Hour11hs	-0.239754	0.161833	-1.481	0.13848	
Hour12hs	-0.572526	0.176261	-3.248	0.00116	**
Hour13hs	-0.866269	0.192619	-4.497	6.88e-06	***
Hour14hs	-1.161169	0.223986	-5.184	2.17e-07	***
Hour15hs	-1.553865	0.243284	-6.387	1.69e-10	***
Hour16hs	-1.748203	0.264167	-6.618	3.65e-11	***
Hour17hs	-1.795306	0.268854	-6.678	2.43e-11	***
TreatmentS:Hour10hs	0.151935	0.217813	0.698	0.48546	
TreatmentS:Hour11hs	0.627543	0.221504	2.833	0.00461	**
TreatmentS:Hour12hs	1.033080	0.230961	4.473	7.71e-06	***
TreatmentS:Hour13hs	1.325415	0.243715	5.438	5.38e-08	***
TreatmentS:Hour14hs	1.632856	0.271808	6.007	1.89e-09	***
TreatmentS:Hour15hs	1.999392	0.285662	6.999	2.58e-12	***
TreatmentS:Hour16hs	2.194687	0.303744	7.225	4.99e-13	***
TreatmentS:Hour17hs	2.339225	0.306555	7.631	2.34e-14	***

Signif. codes: 0 ‘***’ 0.001 ‘**’ 0.01 ‘*’ 0.05 ‘.’ 0.1 ‘ ’ 1

```
sum(residuals(model_hours_bridges,type
="pearson")^2)/df.residual(model_hours_bridges)
[1] 1.104901
```

Contrasts Baseline (9hs) vs. each of the times per treatment

```
***the emmeans1***
meanie1 <- emmeans(model_hours_bridges, pairwise ~ Hour | Treatment)
meanie.contrasts <- contrast(meanie, method = "trt.vs.ctrl", reverse = "true")
print (meanie.contrasts)
```

$emmeans
Values in bold represent p-values<0.05
Signif. codes: 0 ‘***’ 0.001 ‘**’ 0.01 ‘*’ 0.05 ’(*)’ 0.1 ‘NS’ 1

Treatment = B:

contrast   estimate   SE df t.ratio p.value

9hs - 10hs	-0.00549	0.171	87	-0.032	1.0000	NS
9hs - 11hs	0.24885	0.186	87	1.336	0.6387	NS
9hs - 12hs	0.56532	0.202	87	2.805	**0.0407**	*
9hs - 13hs	0.84763	0.219	87	3.874	**0.0016**	**
9hs - 14hs	1.14249	0.239	87	4.773	**0.0001**	***
9hs - 15hs	1.51002	0.271	87	5.569	**<.0001**	***
9hs - 16hs	1.69333	0.291	87	5.816	**<.0001**	***
9hs - 17hs	1.73327	0.295	87	5.877	**<.0001**	***

Treatment = S:

contrast	estimate	SE	df	t.ratio	p.value	
9hs - 10hs	-0.14876	0.183	87	-0.814	0.9071	NS
9hs - 11hs	-0.37470	0.174	87	-2.149	0.1876	NS
9hs - 12hs	-0.44835	0.172	87	-2.608	0.0672	NS
9hs - 13hs	-0.45421	0.172	87	-2.646	0.0612	(*)
9hs - 14hs	-0.16024	0.185	87	-0.865	0.8885	NS
9hs - 15hs	-0.43560	0.173	87	-2.524	0.0824	(*)
9hs - 16hs	-0.43806	0.172	87	-2.540	0.0794	(*)
9hs - 17hs	-0.53470	0.169	87	-3.159	**0.0151**	*

Results are given on the log (not the response) scale.

P value adjustment: dunnettx method for 8 tests

% Change activity with respect to the initial (9hs)

tapply(Activity _bait$Activity, Activity_bait$Time, FUN = mean)

9hs	10hs	11hs	12hs	13hs	14hs	15hs	16hs	17hs
157.5	153.16	132.33	89.66	66.33	47.4	33.66	31.83	31.83

tapply(Activity _sucrose$Activity, Activity_sucrose$Time, FUN = mean)

9hs	10hs	11hs	12hs	13hs	14hs	15hs	16hs	17hs
124.8	142.0	183.3	195.7	196.0	203.4	192.5	192.3	212.2

Bridge Bait

10hs= 2.76 % smaller than initial (9hs)

11hs= 15.98% smaller than initial (9hs)

12hs= 43.07% smaller than initial (9hs)

13hs= 57.89% smaller than initial (9hs)

14hs= 69.9% smaller than initial (9hs)

15hs= 78.63% smaller than initial (9hs)

16hs= 79.8% smaller than initial (9hs)

17hs= 79.8% smaller than initial (9hs)

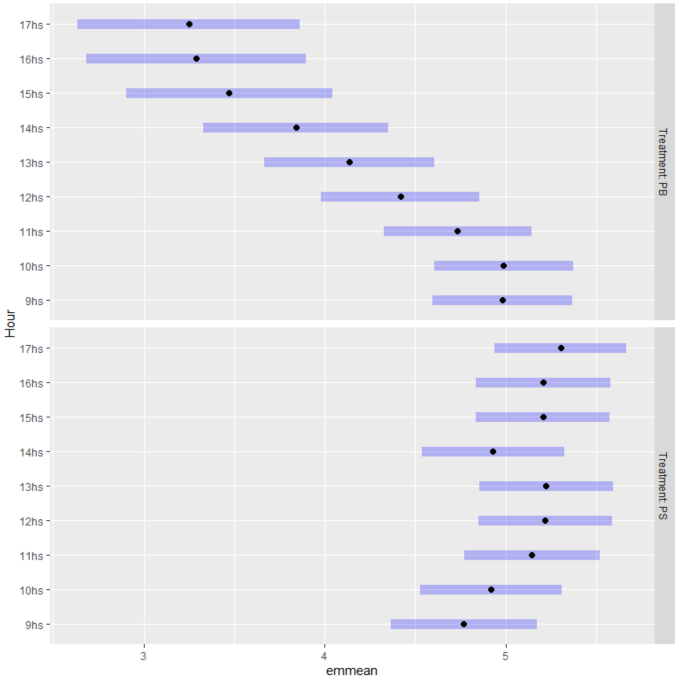

Figure S10. Emmean contrasts of the times within each treatment (PB-PS) in model_hours_bridges

Contrasts between treatment B-S

the emmeans2
meanie2 <- emmeans(model_hour_bridges, pairwise ~ Treatment | Hour)

print (meanie2) $emmeans

$contrasts
Values in bold represent p-values<0.05
Signif. codes: 0 '***' 0.001 '**' 0.01 '*' 0.05 '(*)' 0.1 'NS' 1

Hour = 9hs:

contrast	estimate	SE	df	t.ratio	p.value
B - S	0.2220	0.164	85	1.357	0.1785

Hour = 10hs:

contrast	estimate	SE	df	t.ratio	p.value
B - S	0.0701	0.158	85	0.445	0.6574

Hour = 11hs:

contrast	estimate	SE	df	t.ratio	p.value
**B - S**	**-0.4055**	**0.162**	**85**	**-2.507**	**0.0141**

Hour = 12hs:

contrast	estimate	SE	df	t.ratio	p.value
**B - S**	**-0.8110**	**0.174**	**85**	**-4.653**	**<.0001**

Hour = 13hs:

contrast	estimate	SE	df	t.ratio	p.value
**B - S**	**-1.1034**	**0.191**	**85**	**-5.780**	**<.0001**

Hour = 14hs:

contrast	estimate	SE	df	t.ratio	p.value
**B - S**	**-1.4108**	**0.226**	**85**	**-6.231**	**<.0001**

Hour = 15hs:

contrast	estimate	SE	df	t.ratio	p.value
**B - S**	**-1.7773**	**0.243**	**85**	**-7.328**	**<.0001**

Hour = 16hs:

contrast	estimate	SE	df	t.ratio	p.value
**B - S**	**-1.9726**	**0.264**	**85**	**-7.482**	**<.0001**

Hour = 17hs:

contrast	estimate	SE	df	t.ratio	p.value
**B - S**	**-2.1172**	**0.267**	**85**	**-7.937**	**<.0001**

Results are given on the log (not the response) scale.

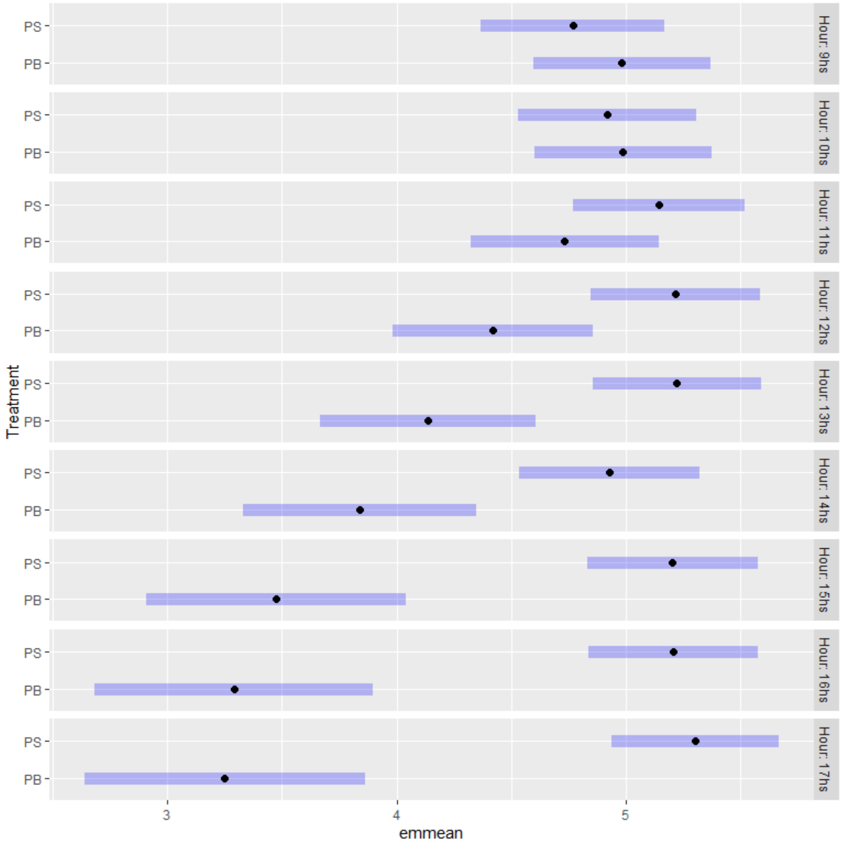

Figure S11. Emmean contrasts between treatments (S-B) at different times (hours) in model_hours_bridges.

V. Trunk Trail Dynamics Over Hours Throughout a Day

The temporal dynamics on the trunk path were evaluated throughout the same day at 3 different times. At 9 am as baseline, and then at 1 and 5 pm. Descriptive graphs were made using ggplot and a linearly generalized mixed model was carried out. The response variable was the number of ants/min in both directions at the sites of the trunk trail closest to the sucrose and the toxicant bridges (0mS and 0m, respectively). The distribution that best fitted the data was the negative binomial. The fixed and qualitative explanatory variables were the sites (0m and 0mS, 2 levels) and time (9am, 1pm and 5pm, 3 levels). Note that we chose to consider time as a factor, rather than a continuous variable, in order to facilitate pairwise comparisons. Replicates were considered as a random explanatory variable (n = 6). A linearly generalized mixed model with interaction was carried out.

Activity = Sites* Hours + (random effects: Replica), distribution family: negative binomial (log link function).

Pairwise comparisons of activity between treatments were conducted using the emmeans:contrast() function.

Results:

In this experiment, we measured activity on the trunk trail specifically at the points where the bridges made contact with the trail. We conducted measurements at three different time points (baseline and 2 more times: 4 and 8 hours after toxic bait access).

There is an interaction between the time and the sites: Activity at the location in contact with the control bridge did not differ from its baseline at any time. By contrast, activity at the location close to the toxicant bridge changed with the time. Specifically, it showed a decrease after 4 hours of bait consumption (baseline-4h: estimate = 0.75; p = 0.002). 8 hours after bait consumption the activity did not differ from baseline (baseline - 8h: estimate = 0.59; p = 0.16. Fig. S12).

This again confirms that abandonment is initiated in a very localised manner in the vicinity of the toxic bait. Furthermore, it also proves that the abandonment is not driven by a reduction in the population, as the activity levels at the trunk trails remained high.

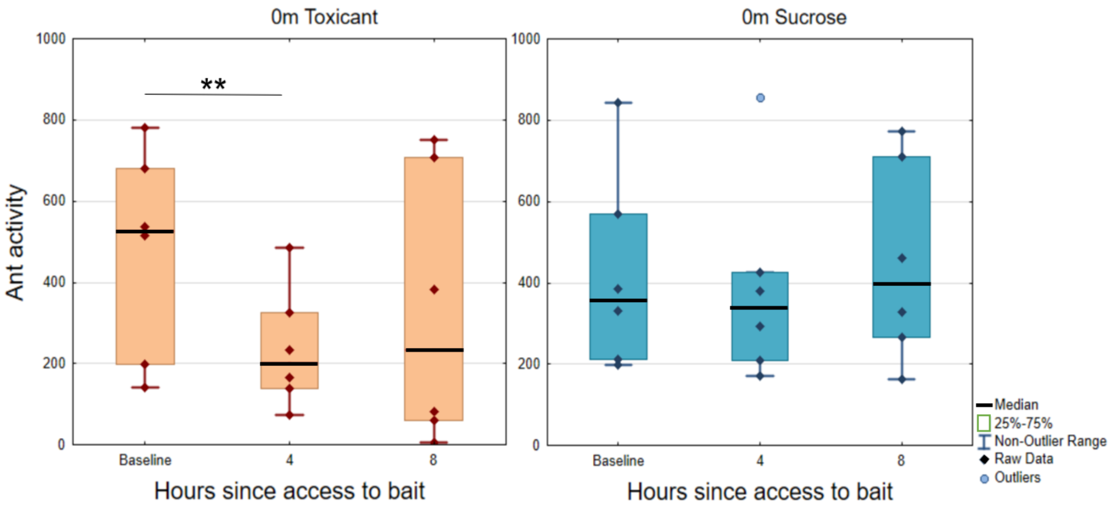

Figure S12: Ant activity on the trunk trail; temporal dynamics in hours. Activity measured at two locations at the trunk trail: by a bridge offering a toxicant (orange. 0m Toxicant bridge), and by a control bridge offering unadulterated sucrose (blue. 0m Sucrose bridge) placed over 7 meters away. Activity was measured three times: at 9 am when both bridges still offered sugar (baseline for each bridge). Immediately thereafter, the feeders were opened, offering sucrose solution in one bridge and the toxic bait in the toxicant bridge. Then activity on the trunk trail was measured again at 4 and 8 hours later. Each of those times were compared with the respective baseline; (**: $p<0.01$; No symbol: not significant).

Details of the Model for trunk trail over hours

Response Variable: Activity (mean count of ants/min)

Site: 0m Suc (SS) – 0m Tox (SB). *2 levels* (2 sites, where each bridge was in contact with the trunk trail)

Time (hours): (Baseline) – (time 4) – (time 8). *3 levels*

Replica: n=6

. **Figure S13.** Residuals vs. Predicted to validate Model_trunk_hours using Dharma

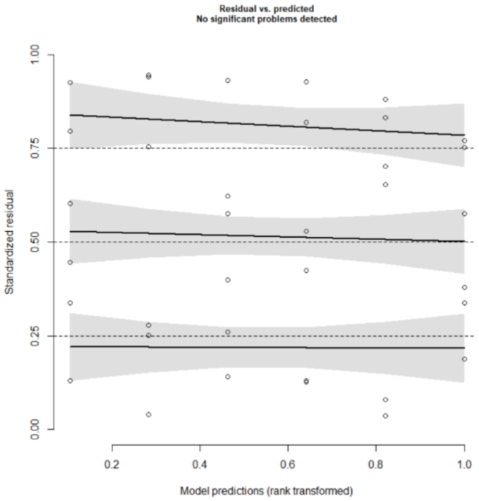

Set of variables considered in the generalized linear (mixed) model trunk hours.

model_trunk_hours <- glmmTMB(Activity ~ Sites*Hours + (1|Replica), dispformula = ~ Hours, family = nbinom1(link = "log"), data = trunk_hour)

summary(model_trunk_hours)

```
Family: nbinom1  ( log )
Formula:          Activity ~ Sites * Hours + (1 | Replica)
Dispersion:              ~ Hours
Data: trunk_hour

     AIC      BIC   logLik deviance df.resid
   485.4    501.2   -232.7    465.4       26

Random effects:

Conditional model:
 Groups  Name        Variance Std.Dev.
 Replica (Intercept) 0.2497   0.4997
Number of obs: 36, groups:  Replica, 6

Conditional model:
                 Estimate Std. Error z value Pr(>|z|)
(Intercept)        6.0469     0.2174  27.821  < 2e-16 ***
```

```
SitesSS          -0.1140     0.1022  -1.115 0.264755    
Hours13hs          -0.7480     0.2044  -3.659 0.000253 ***
Hours17hs          -0.5870     0.3356  -1.749 0.080323 .  
SitesSS:Hours13hs   0.6969     0.2575   2.707 0.006790 ** 
SitesSS:Hours17hs   0.7897     0.3955   1.997 0.045853 *  
---
Signif. codes:  0 ‘***’ 0.001 ‘**’ 0.01 ‘*’ 0.05 ‘.’ 0.1 ‘ ’ 1

Dispersion model:
            Estimate Std. Error z value Pr(>|z|)    
(Intercept)   2.5723     0.6874   3.742 0.000183 ***
Hours13hs     1.3592     0.8419   1.614 0.106428    
Hours17hs     2.7417     0.8214   3.338 0.000844 ***
---
Signif. codes:  0 ‘***’ 0.001 ‘**’ 0.01 ‘*’ 0.05 ‘.’ 0.1 ‘ ’ 1
```

Contrasts Baseline (9hs) vs. each of the times per treatment

```
meanie <- emmeans(model_trunk_hours, pairwise ~ Hours | Sites)
meanie.contrasts <- contrast(meanie, method = "trt.vs.ctrl", reverse = "true")
print (meanie.contrasts)
```

$emmeans

Values in bold represent p-values<0.05
Signif. codes: 0 ‘***’ 0.001 ‘**’ 0.01 ‘*’ 0.05 ’(*)’ 0.1 ‘NS’ 1

Sites = SB:

contrast	estimate	SE	df	t.ratio	p.value	
9hs - 13hs	0.7480	0.204	26	3.659	**0.0022**	**
9hs - 17hs	0.5870	0.336	26	1.749	0.1657	

Sites = SS:

contrast	estimate	SE	df	t.ratio	p.value
9hs - 13hs	0.0511	0.163	26	0.314	0.9183
9hs - 17hs	-0.2028	0.256	26	-0.792	0.6458

Results are given on the log (not the response) scale.

P value adjustment: dunnettx method for 2 tests

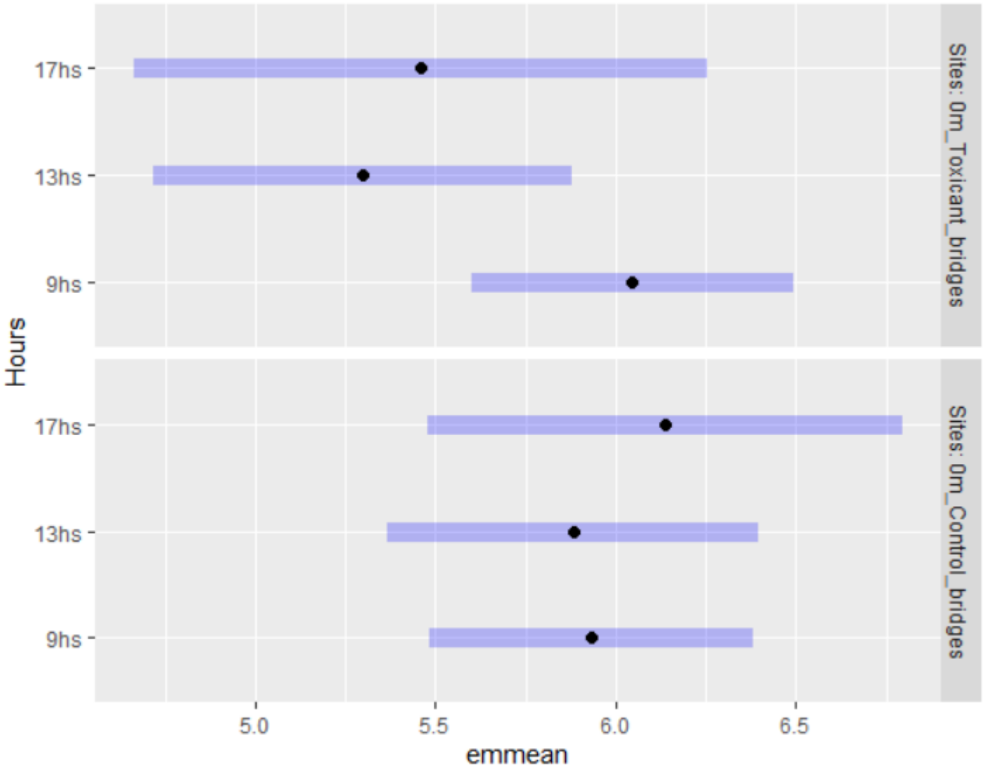

Figure S14. Emmean contrasts of the times within each of the two sites (0m Tox and 0m Suc) in model_trunk_hours.

In this experiment, the trunk trail near the Toxic Bridge was impacted at 4 h after bait access, whereas in the previous experiment at 18-20 hours, the trunk trail remained unaffected. At the following time (i.e., 8 h after bait access) the variability was so high that no significance was found. Why was the activity in the trunk trail affected more rapidly than in the previous experiment in this case? The higher initial activity on the bridges during bait activation likely caused a larger influx of toxic bait in the initial hours after bait access. Then, the difference between experiments might be explained by a greater early intake of toxic bait results in stronger and faster abandonment effects and over a broader area. Supporting this argument, the toxic bridge's replica with the highest initial activity showed a more abrupt decrease in activity along the trunk path after 8 hours of accessing the bait. From 780 individuals per minute at time 0, it dropped down within 4 hours to 139 and further to only 61 ants passing near the toxic bridge after 8 hours. Meanwhile, just 7 meters away by the sucrose bridge, a reduction in activity didn't occur; it remained consistent during those time intervals, with 843, 857, and 772 ants passing per minute respectively.

However, the initial quantity likely relates to the size of the colony in which the bait is distributed. If the bait is returned to a small colony or colony fragment, the impact might be quicker and more pronounced compared to the same influx distributed among a much larger group.

VI. Assessing Mortality

Mortality tests were carried out on different days and using ants from 3 different nests. In total, mortality was analysed in 24 containers per treatment (sucrose solution or toxic bait) for 6 hours, observing the number of live ants per hour.

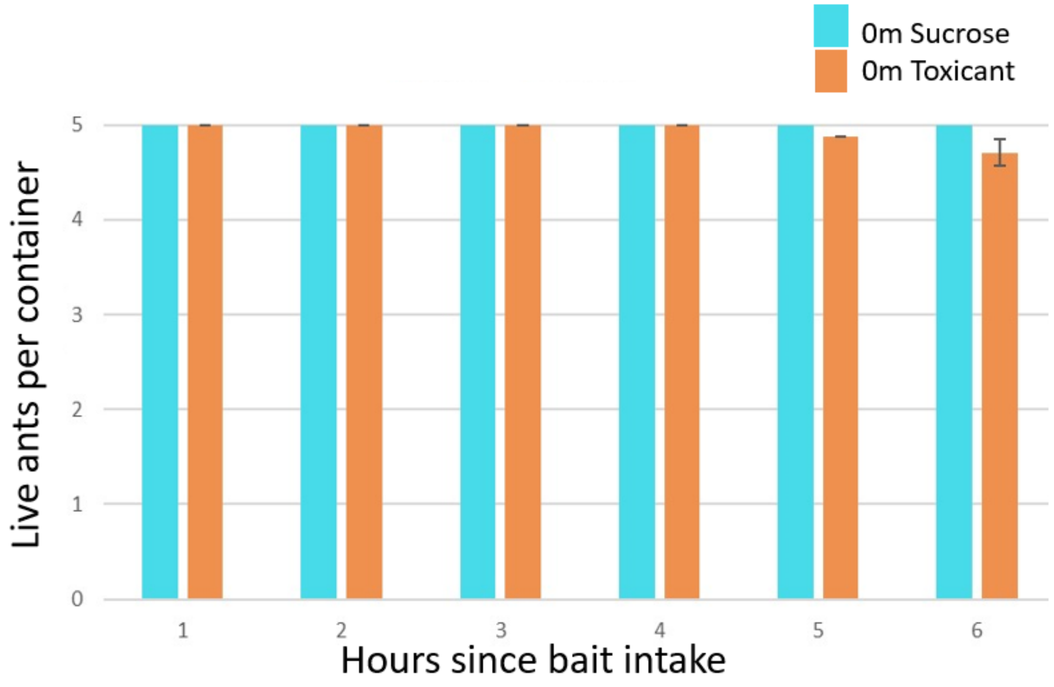

Figure S15. Live ants per container (mean ± SE) in the sucrose (cyan; N = 24 containers) and toxic bait (orange; N = 24 containers) treatments during the 6 hours of the mortality test. Each container had 5 ants.

VII. Supplementary References

Brooks M, Bolker B, Kristensen K, Maechler M, Magnusson A, McGillycuddy M, Skaug H, Nielsen A, Berg C, Bentham Kv, Sadat N, Lüdecke D, Lenth R, O'Brien J,

Geyer CJ, Jagan M, Wiernik B, Stouffer DB, 2023. Package ‘glmmTMB’. Generalized Linear Mixed Models using Template Model Builder. V 117.
Hartig F, 2020. DHARMa: Residual diagnostics for hierarchical (multi-level / mixed) regression models. R package version 031 https://florianhartig.github.io/DHARMa/: florianhartig.
Hothorn T, Bretz F, Westfall P, Heiberger RM, Schuetzenmeister A, Scheibe S, 2023. Simultaneous Inference in General Parametric Models. R Core Team.
Pinheiro J, Bates D, DebRoy S, Sarkar D, Heisterkamp S, Willigen BV, Ranke J, 2023. Package ‘nlme’. Linear and Nonlinear Mixed Effects Models. R Core Team <R-core@R-project.org>.
Searle SR, Speed FM, Milliken GA, 1980. Population marginal means in the linear model: an alternative to least squares means. The American Statistician 34:216-221. doi: 10.1080/00031305.1980.10483031.
Wickham MH, 2014. ggplot2. An implementation of the grammar of graphics. Package “ggplot2”.
