# Supplementary material for: Ants evade harmful food by active abandonment
Source: Commun Biol. 2024 Jan 12;7:84. doi: 10.1038/s42003-023-05729-7 (PMC10786876; doi:10.1038/s42003-023-05729-7)
Supplement: Supplementary file 2 — Reporting Summary [file 42003_2023_5729_MOESM2_ESM.pdf]

nature portfolio

Corresponding author(s): Roxana Josens
Last updated by author(s): Dec 14, 2023

Reporting Summary

Nature Portfolio wishes to improve the reproducibility of the work that we publish. This form provides structure for consistency and transparency in reporting. For further information on Nature Portfolio policies, see our Editorial Policies and the Editorial Policy Checklist.

Statistics

For all statistical analyses, confirm that the following items are present in the figure legend, table legend, main text, or Methods section.

n/a	Confirmed	
☐	☒	The exact sample size (*n*) for each experimental group/condition, given as a discrete number and unit of measurement
☐	☒	A statement on whether measurements were taken from distinct samples or whether the same sample was measured repeatedly
☐	☒	The statistical test(s) used AND whether they are one- or two-sided<br>*Only common tests should be described solely by name; describe more complex techniques in the Methods section.*
☐	☒	A description of all covariates tested
☐	☒	A description of any assumptions or corrections, such as tests of normality and adjustment for multiple comparisons
☐	☒	A full description of the statistical parameters including central tendency (e.g. means) or other basic estimates (e.g. regression coefficient) AND variation (e.g. standard deviation) or associated estimates of uncertainty (e.g. confidence intervals)
☐	☒	For null hypothesis testing, the test statistic (e.g. *F*, *t*, *r*) with confidence intervals, effect sizes, degrees of freedom and *P* value noted<br>*Give P values as exact values whenever suitable.*
☒	☐	For Bayesian analysis, information on the choice of priors and Markov chain Monte Carlo settings
☒	☐	For hierarchical and complex designs, identification of the appropriate level for tests and full reporting of outcomes
☒	☐	Estimates of effect sizes (e.g. Cohen's *d*, Pearson's *r*), indicating how they were calculated

Our web collection on statistics for biologists contains articles on many of the points above.

Software and code

Policy information about availability of computer code

Data collection	No software was used.
Data analysis	Statistical analyses were performed in R Studio using different packages (glmmTMB, nlme, and multcomp, etc.)

For manuscripts utilizing custom algorithms or software that are central to the research but not yet described in published literature, software must be made available to editors and reviewers. We strongly encourage code deposition in a community repository (e.g. GitHub). See the Nature Portfolio guidelines for submitting code & software for further information.

Data

Policy information about availability of data

All manuscripts must include a data availability statement. This statement should provide the following information, where applicable:

- Accession codes, unique identifiers, or web links for publicly available datasets
- A description of any restrictions on data availability
- For clinical datasets or third party data, please ensure that the statement adheres to our policy

All data will be fully available under the following link: https://figshare.com/s/18abcfa3e89b5dd68d00

Research involving human participants, their data, or biological material

Policy information about studies with human participants or human data. See also policy information about sex, gender (identity/presentation), and sexual orientation and race, ethnicity and racism.

Reporting on sex and gender	*Use the terms sex (biological attribute) and gender (shaped by social and cultural circumstances) carefully in order to avoid confusing both terms. Indicate if findings apply to only one sex or gender; describe whether sex and gender were considered in study design; whether sex and/or gender was determined based on self-reporting or assigned and methods used. Provide in the source data disaggregated sex and gender data, where this information has been collected, and if consent has been obtained for sharing of individual-level data; provide overall numbers in this Reporting Summary. Please state if this information has not been collected. Report sex- and gender-based analyses where performed, justify reasons for lack of sex- and gender-based analysis.*
Reporting on race, ethnicity, or other socially relevant groupings	*Please specify the socially constructed or socially relevant categorization variable(s) used in your manuscript and explain why they were used. Please note that such variables should not be used as proxies for other socially constructed/relevant variables (for example, race or ethnicity should not be used as a proxy for socioeconomic status). Provide clear definitions of the relevant terms used, how they were provided (by the participants/respondents, the researchers, or third parties), and the method(s) used to classify people into the different categories (e.g. self-report, census or administrative data, social media data, etc.) Please provide details about how you controlled for confounding variables in your analyses.*
Population characteristics	*Describe the covariate-relevant population characteristics of the human research participants (e.g. age, genotypic information, past and current diagnosis and treatment categories). If you filled out the behavioural & social sciences study design questions and have nothing to add here, write "See above."*
Recruitment	*Describe how participants were recruited. Outline any potential self-selection bias or other biases that may be present and how these are likely to impact results.*
Ethics oversight	*Identify the organization(s) that approved the study protocol.*

Note that full information on the approval of the study protocol must also be provided in the manuscript.

Field-specific reporting

Please select the one below that is the best fit for your research. If you are not sure, read the appropriate sections before making your selection.

☒ Life sciences ☐ Behavioural & social sciences ☐ Ecological, evolutionary & environmental sciences

For a reference copy of the document with all sections, see nature.com/documents/nr-reporting-summary-flat.pdf

Life sciences study design

All studies must disclose on these points even when the disclosure is negative.

Sample size	We did not perform a formal sample size calculation. For each replicate, it was essential to locate a high-activity ant path with approximately 12 linear meters of horizontal exposure. Due to the difficulty of this task, we were only able to achieve 5 to 6 repetitions in each experiment.
Data exclusions	In one replica of Experiment 2 (Foraging Trail Dynamics Over Hours), one bridge experienced a brief temperature spike exceeding 40°C at 2 p.m., resulting in the absence of ants on this bridge during that specific time point. Subsequently, as the temperature dropped slightly in the next recording, ant activity resumed at previous levels. Consequently, data from both bridges in that particular replicate during that time point were excluded from the analysis.
Replication	Our replications were conducted on different trunk trails and across different years, consistently showing a decrease in activity at the toxic food bridge while observing either the maintenance or an increase in activity at the sucrose food bridge. Pilot tests performed during the prior year also yielded the same results.<br><br>As discussed in the manuscript, the dynamics of this behavior may vary in different scenarios. Field conditions, such as population size, the presence of alternative food sources, and the initial number of ants accessing the bait, are likely to influence these variations. While there may be instances of temporary spikes in activity on the toxicant bridge, they tend to subside quickly again. Therefore, in average the results are strong and replicable.
Randomization	The path selection was non-random (it was based on specific criteria: path length, activity level and the same horizontal substrate for the trail). Additionally, the locations of the two bridges within the trail were pre-determined. The Toxic food bridge was positioned near the midpoint to allow measurements on both sides of the trunk trail, while the Sucrose bridge was consistently placed more than 6 m away from the other bridge.
Blinding	Blind counting was not feasible due to the same experimenter was involved in the entire process, from bridge placement and experimentation to video recording and ant counting from the video. The experimenter's familiarity with the images made it impractical to maintain blindness, as he could identify the specific bridge in question.

Reporting for specific materials, systems and methods

We require information from authors about some types of materials, experimental systems and methods used in many studies. Here, indicate whether each material, system or method listed is relevant to your study. If you are not sure if a list item applies to your research, read the appropriate section before selecting a response.

Materials & experimental systems

n/a	Involved in the study
☒	☐ Antibodies
☒	☐ Eukaryotic cell lines
☒	☐ Palaeontology and archaeology
☐	☒ Animals and other organisms
☒	☐ Clinical data
☒	☐ Dual use research of concern
☒	☐ Plants

Methods

n/a	Involved in the study
☒	☐ ChIP-seq
☒	☐ Flow cytometry
☒	☐ MRI-based neuroimaging

Animals and other research organisms

Policy information about studies involving animals; ARRIVE guidelines recommended for reporting animal research, and Sex and Gender in Research

Laboratory animals	Linepithema humile ants.
Wild animals	We filmed ant trails with cell phones and a camera. All of the trails were from Linepithema humile.
Reporting on sex	For all the ant species, workers are female.
Field-collected samples	For the laboratory test, we used Linepithema humile nests (with queens and more than 2000 individuals) obtained in the campus of the University of Buenos Aires, at least a year before the experiment was performed. The artificial nests consisted of plastic boxes (30x50x30 cm3) with bottoms made of plaster and a central hollow covered by a glass plate. Walls were fluon painted to prevent ants from escaping. The colonies were kept in the laboratory in a natural light/dark cycle at nearly constant temperature (25+/-3 °C). Ants could move freely within the nest and had access to a water source. Between experiments, ants were fed with honey–water and chopped cockroaches.
Ethics oversight	No ethical approval was required.

Note that full information on the approval of the study protocol must also be provided in the manuscript.

Plants

Seed stocks	*Report on the source of all seed stocks or other plant material used. If applicable, state the seed stock centre and catalogue number. If plant specimens were collected from the field, describe the collection location, date and sampling procedures.*
Novel plant genotypes	*Describe the methods by which all novel plant genotypes were produced. This includes those generated by transgenic approaches, gene editing, chemical/radiation-based mutagenesis and hybridization. For transgenic lines, describe the transformation method, the number of independent lines analyzed and the generation upon which experiments were performed. For gene-edited lines, describe the editor used, the endogenous sequence targeted for editing, the targeting guide RNA sequence (if applicable) and how the editor was applied.*
Authentication	*Describe any authentication procedures for each seed stock used or novel genotype generated. Describe any experiments used to assess the effect of a mutation and, where applicable, how potential secondary effects (e.g. second site T-DNA insertions, mosiacism, off-target gene editing) were examined.*
